# Regional differences in healthcare costs further explained: The contribution of health, lifestyle, loneliness and mastery

**DOI:** 10.1007/s12508-022-00369-4

**Published:** 2022-11-02

**Authors:** Rachelle Meisters, Daan Westra, Polina Putrik, Hans Bosma, Dirk Ruwaard, Maria Jansen

**Affiliations:** 1 Department of Health Services Research, Care and Public Health Research Institute (CAPHRI), Faculty of Health, Medicine and Life Sciences (FHML), Maastricht University, Maastricht, The Netherlands; 2 Academische Werkplaats voor Publieke Gezondheid Limburg, GGD Zuid Limburg, Heerlen, The Netherlands; 3 Department of Social Medicine, Care and Public Health Research Institute (CAPHRI), Faculty of Health, Medicine and Life Sciences (FHML), Maastricht University, Maastricht, The Netherlands

**Keywords:** Regional health inequalities, Healthcare costs, Lifestyle, Loneliness, Mastery

## Abstract

Healthcare costs in the Netherlands are rising and vary considerably among regions. Explaining regional differences in healthcare costs can help policymakers in targeting appropriate interventions in order to restrain costs. Factors usually taken into account when analyzing regional differences in healthcare costs are demographic structure and socioeconomic status (SES). However, health, lifestyle, loneliness and mastery have also been linked to healthcare costs. Therefore, this study analyzes the contribution of health, lifestyle factors (BMI, alcohol consumption, smoking and physical activity), loneliness, and mastery to regional differences in healthcare costs. Analyses are performed in a linked dataset (*n* = 334,721) from the Dutch Public Health Services, Statistics Netherlands, the National Institute for Public Health and the Environment (year 2016), and the healthcare claims database Vektis (year 2017) with Poisson and zero-inflated binomial regressions. Regional differences in general practitioner consult costs remain significant even after taking into account health, lifestyle, loneliness, and mastery. Regional differences in costs for mental, pharmaceutical, and specialized care are less pronounced and can be explained to a large extent. For total healthcare costs, regional differences are mostly explained through the factors included in this study. Hence, addressing lifestyle factors, loneliness and mastery can help policymakers in restraining healthcare costs. In this study, the region of Zuid-Limburg represents the reference region. Use compare regions for health and healthcare costs (Regiovergelijker gezondheid en zorgkosten) in order to select all other Dutch regions as reference region.

**Supplementary Information:**

The online version of this article (10.1007/s12508-022-00369-4) contains supplementary material, which is available to authorized users.

## Introduction

Healthcare costs in the Netherlands continue to increase annually [1]. Before the coronavirus pandemic, the average annual growth for the coming decades was calculated to be 2.8%, according to the Dutch National Institute for Public Health and the Environment [1]. With rising healthcare costs, the affordability of the Dutch healthcare system is under increasing pressure. In addition, average healthcare costs vary strongly between regions and municipalities in the Netherlands. In 2018, the average healthcare costs, as reimbursed by the basic health insurance plan, averaged € 2,625 per insured year (insured years are insured persons weighted for the registration period in the particular municipality; this makes it possible to compare municipalities regardless of births, deaths, or relocations). Between the municipalities with the highest and lowest healthcare costs, however, there was a difference of more than € 1,700 (for example, € 3,625 in Kerkrade and € 1,853 in Urk). Even after adjusting for age and gender, the difference between the municipalities with the highest and lowest healthcare costs was approximately € 1,200 (for example, € 3,273 for Heerlen and € 2,074 for Rozendaal) [2]. The regions with higher healthcare costs are the border regions of Zeeland, Groningen, Drenthe, and South Limburg (*Zuid-Limburg*) in particular. In order to reduce the increase in healthcare costs, explanations for regional differences are needed to provide insight into possible leads.

A study based on relocations in the Netherlands between 2006 and 2013 showed that 70% of regional differences in total healthcare costs are explained by demand factors [3]. Demand factors are all factors that pertain to the individual, such as level of education and health status. In a previous study on population funding, regional differences in specific groups of the chronically ill (people with depression and diabetics) were largely explained by demand factors [4]. In these healthcare cost studies, a part of the Dutch population was assessed, adjusted for age and gender [3, 4], socioeconomic status (SES), and self-reported health [4]. In addition to demographic characteristics, SES, and self-reported health [5], there are other factors that appear to play a role in higher healthcare utilization and, as a result, higher costs, such as an unhealthy lifestyle [6, 7] and loneliness [8, 9]. Lonely citizens visit a doctor more often [8, 9], are more frequently in need of mental healthcare [8] and inpatient care, and are more likely to take antidepressants and anxiolytics [9].

In this national study, we aimed to explain regional variations in healthcare costs based on lifestyle factors, loneliness, and mastery, after adjusting for demographic factors, SES, and general and mental health. In an accompanying article in a special issue of the *Journal for Health Sciences* (*Tijdschrift voor gezondheidswetenschappen*), we also show that lifestyle, loneliness, and mastery partly explain regional differences in general and mental health (operationalized by means of self-perceived health, chronic illness, and psychological distress) [10].

The research question for the current study was: “Do lifestyle factors, loneliness, and mastery contribute to explaining regional differences in healthcare costs in addition to age, gender, SES, and health status?” The results can direct future preventive interventions and policies to help reduce regional differences in healthcare costs.

## Methods

### Data and sampling

Data were extracted from a linked dataset provided by the Dutch Public Health Services, Statistics Netherlands, and the healthcare claims–based database Vektis. The Health Survey 2016 provides information about demographic factors, SES, lifestyle, loneliness, mastery, and physical and mental state of health of the respondents [10]. Registry data from Statistics Netherlands (based on the Dutch Personal Records Database and the Dutch Tax and Customs Administration) were linked to the survey data. This linkage provided more information about the migration background of respondents and their household income. Finally, data on national healthcare claims (covered by the basic health insurance under the Healthcare Insurance Act) provided by Vektis were linked for the year 2017.

The datasets were linked using pseudonymized RIN numbers provided by Statistics Netherlands in a secured digital environment. After data linkage and exclusion of missing data, the sample consisted of 334,721 persons. To ensure the representativeness of the sample, weighting factors were added to the Health Survey [10].

### Dependent variables

The dependent variables were the following five cost categories: total costs (all costs reimbursed by the Healthcare Insurance Act), general practitioner (GP) consultation costs, pharmaceutical costs, specialized care costs (hospital and curative care), and mental healthcare costs (inpatient and outpatient mental healthcare and long-term mental healthcare). For inpatient mental healthcare, costs are covered by the Healthcare Insurance Act for up to three years. After three years, these costs are covered by the Dutch Long-term Care Act. The costs covered by the Long-Term Care Act were not included in the Vektis data.

### Independent variables

The independent variables were region, demographic characteristics, SES, general and mental health, lifestyle, loneliness, and mastery. For the variable “region”, we used the regional classification of the Public Health Survey 2016, with the region of Zuid-Limburg as the reference group. Zuid-Limburg was chosen because this is the region with the highest healthcare costs, the highest number of adults with a chronic disease [11], the lowest percentage of adults with a good self-rated health [12], the highest number of lonely adults [13], and the highest number of adults with insufficient mastery [14].

Demographic factors were age, gender, migration background (“Dutch”, “Western migration background”, or “non-Western migration background”), and marital status (“married/living together”, “never married”, “widowed”, or “divorced”). SES consisted of the highest attained level of education (“primary education”, “lower vocational education”, “middle vocational or secondary education”, or “higher vocational education or university”), standardized household income quartile, and income inadequacy (“inadequate, major concerns”, “inadequate, some concerns”, “adequate, minor concerns”, or “adequate, no concerns”) [10]. To assess general health, self-rated health (“(very) good” or “fair, (very) poor”) and self-reported chronic disease (“yes, one or more” or “no”) were used. The variable “psychological distress” (assessed with the Kessler-10 questionnaire [15]) was used as a proxy for the mental component of health, resulting in a total score ranging from 10 to 50 (a score of 10–29 indicated “no or low risk”, and a score of ≥ 30 indicated “high risk”). Lifestyle factors included body mass index category (< 18.5 kg/m^2^ was considered “underweight”, 18.5–25 kg/m^2^ was “normal”, 25–30 kg/m^2^ was “overweight”, and > 30 kg/m^2^ was “obese”), smoking history (“never”, “former smoker”, or “current smoker”), alcohol consumption (“never”, “moderate”, or “excessive”) and (in)sufficient physical activity. Loneliness was assessed with the De Jong-Gierveld scale [16] (continuous score ranging from 0 to 11, whereby a score of 3–8 was considered “moderate”, 9–10 was “severe”, and 11 was “very severe loneliness”). Mastery was assessed with the Pearlin Mastery Scale (continuous score ranging from 7 to 35, whereby a score ≤ 19 was considered “insufficient mastery”) [17].

### Statistical analyses

For total healthcare costs, the incidence rate ratio (IRR) was assessed with Poisson regressions. The IRR represented the change in costs per change of the particular independent variable. For the cost categories of GP consultations, pharmacy, specialized care, and mental healthcare, zero-inflated binomial (ZINB) distributions were used, because most people do not need all these types of healthcare and these cost data therefore contain many zeros (Tab. 1). The ZINB model is a combination of two separate models. The first part is a logistics model that estimates the probability that a person incurs no costs. The second model is a negative binomial model, which modulates the amount of costs for those who do incur costs. The ZINB model yields two sets of estimates: the odds ratio (OR) for the logistic model and the IRR for the negative binomial model.

**Table 1:** Descriptive data for healthcare costs based on unweighted data

	total sample (*n* = 334,721)			respondents with costs			
cost category	Median (IQR)	Q1	Q3	*n* (%)	Median (IQR)	Q1	Q3
*total*^*a*^	€ 814.78 (€ 2,361.94)	€ 220.17	€ 2,582.11	334,276 (99.9%)	€ 817.44 (€ 2,364.87)	€ 221.45	€ 2,586.32
*GP consultation*^*a*^	€ 31.55 (€ 56.30)	€ 9.07	€ 65.37	276,873 (82.7%)	€ 40.80 (€ 58.00)	€ 18.62	€ 76.62
*mental healthcare*^*a*^	€ 0.00 (€ 0.00)	€ 0.00	€ 0.00	10,946 (3.3%)	€ 1,323.88 (€ 2,643.87)	€ 805.69	€ 3,449.56
*pharmaceutical care*^*a*^	€ 96.65 (€ 337.69)	€ 15.27	€ 352.96	267,385 (79.9%)	€ 161.87 (€ 421.20)	€ 54.08	€ 475.28
*specialized care*^*a*^	€ 217.86 (€ 1,155.40)	€ 0.00	€ 1155.40	244,774 (63.1%)	€ 576.23 (€ 1,725.76)	€ 144.13	€ 1,869.89

*IQR* interquartile range, *Q1* lowest quartile, *Q3* highest quartile, *GP* general practitioner

^a^Vektis data

For Model 1, only region was included. In Model 2, the demographic characteristics and SES were added to region. Self-perceived health was added in Model 3a, self-reported chronic disease in Model 3b, and psychological distress in Model 3c. In Model 4, the correlation of region with healthcare costs was corrected for demographic factors, SES, self-perceived health, chronic illness, and psychological distress. Lifestyle was added in Model 5a, loneliness in Model 5b, and mastery in Model 5c. In Model 6, all beforementioned factors were included.

Marginal average costs were calculated based on the unadjusted costs (Model 1) and the adjusted costs (Model 6). Marginal costs per region represented the average costs per person in a particular region if the population of this region shared the same demographic factors, SES, general and mental health, lifestyle, loneliness, and mastery as the entire Dutch population. All analyses were performed in Stata 15 [18]. Multiple data imputation to account for missing data was considered. However, in Stata, ZINB models cannot be performed with data imputation, and the analyses were therefore performed with complete data. From previous analyses with this dataset, it appeared that the results of the complete data (complete case analyses) were comparable with the results of the multiple imputed data [10], which indicated that the missing completely at random assumption could be made. Therefore, only the complete case sample was used.

## Results

Slightly over half of the sample was female (52.4%), and the mean age was 59.2 years (standard deviation (SD): 16.9) (Appendix Table A1; [10]). The majority of the respondents reported a (very) good health (74.0%) and no chronic disease (60.7%), whereas 4.5% of the respondents reported psychological distress. With regard to healthcare costs in 2017, 17.3% of the respondents incurred no GP consultation costs, 20.1% did not incur any pharmaceutical costs, 26.9% did not incur any specialized care costs, and 96.7% of the respondents incurred no mental healthcare costs (Tab. 1). The percentages of missing data per variable are shown in Appendix Table A2.

The results for total healthcare costs in the unadjusted model (Model 1) and the most comprehensive model (Model 6) are shown in Tab. 2. For total healthcare costs, most regional differences with Zuid-Limburg could be explained. Residents of 22 of the other 24 regions incurred significantly lower healthcare costs compared with residents of Zuid-Limburg in the unadjusted model; the IRR varied from 0.77 to 0.90 (Tab. 2). In the most comprehensive model, residents of Northern Holland (*Hollands Noorden*) (IRR: 0.93) and South Holland–South (*Zuid-Holland-Zuid*) (IRR: 0.92) incurred significantly lower total healthcare costs than residents of Zuid-Limburg. Residents of the region of Amsterdam incurred significantly higher healthcare costs (IRR: 1.14) (Tab. 2).

**Table 2:** Incidence rate ratios per region for total healthcare costs compared with Zuid-Limburg based on Poisson regressions (*n* = 334,721)

	IRR (95% CI)	
region	Model 1 (region)	Model 6 (total)
Zuid-Limburg	1.00 (ref)	1.00 (ref)
Zuid-Holland-Zuid	0.78 (0.71–0.85)	0.92 (0.86–0.99)
Zeeland	0.91 (0.84–0.99)	1.02 (0.95–1.09)
Zaanstreek-Waterland	0.86 (0.80–0.93)	1.00 (0.93–1.06)
West-Brabant	0.89 (0.82–0.95)	1.04 (0.97–1.11)
Utrecht	0.79 (0.74–0.84)	1.01 (0.96–1.08)
Twente	0.89 (0.80–0.99)	1.08 (0.97–1.20)
Rotterdam-Rijnmond	0.90 (0.84–0.96)	1.02 (0.96–1.09)
Noord- en Oost-Gelderland	0.84 (0.79–0.90)	0.98 (0.92–1.04)
Limburg-Noord	0.93 (0.86–1.01)	1.03 (0.95–1.11)
Kennemerland	0.82 (0.76–0.88)	1.03 (0.96–1.10)
IJsselland	0.84 (0.77–0.91)	1.02 (0.95–1.10)
Hollands Noorden	0.77 (0.72–0.83)	0.93 (0.87–0.99)
Hollands Midden	0.80 (0.75–0.86)	0.95 (0.89–1.01)
Hart voor Brabant	0.85 (0.76–0.94)	1.02 (0.92–1.13)
Haaglanden	0.90 (0.83–0.97)	1.06 (0.98–1.14)
Groningen	0.81 (0.75–0.88)	0.98 (0.91–1.06)
Gooi en Vechtstreek	0.85 (0.77–0.94)	1.00 (0.91–1.10)
Gelderland-Zuid	0.87 (0.79–0.96)	1.06 (0.96–1.17)
Gelderland-Midden	0.82 (0.76–0.90)	0.97 (0.90–1.05)
Friesland	0.85 (0.78–0.91)	1.00 (0.93–1.07)
Flevoland	0.78 (0.69–0.87)	0.96 (0.86–1.06)
Drenthe	0.85 (0.77–0.95)	0.99 (0.89–1.09)
Brabant-Zuidoost	0.87 (0.81–0.93)	1.02 (0.96–1.09)
Amsterdam	0.86 (0.79–0.94)	1.14 (1.04–1.25)

Model 1 only takes region into account. In Model 6, the association between region and healthcare costs was corrected for demographic factors, socioeconomic status (*SES*), general and mental health, lifestyle, loneliness, and mastery. Registry data: age, gender, migration background, and household income. Self-reported data: marital status, education, income inadequacy, general and mental health, lifestyle, loneliness, and mastery

*IRR* incidence rate ratio, *CI* confidence interval

The results for GP consultation, mental healthcare, pharmaceutical, and specialized care costs are twofold and are shown in Appendix Tables A4–A7. In addition, the marginal costs were calculated based on both parts of the ZINB model. These are visualized in Appendix Figures A1–A4.

By adjusting for demographic factors, SES, and general and mental health, the observed cost differences between regions could be largely explained. The differences that persisted after these corrections could be partly explained by lifestyle, loneliness, and mastery (Appendix Tables A3–A7). Lifestyle (Model 5a) mainly contributed to (small) regional differences in total, GP consultation, pharmaceutical, and specialized care costs. Loneliness (Model 5b) contributed to explaining the (small) regional differences in GP consultation, mental healthcare, and specialized care costs. Mastery (Model 5c) contributed to the (small) differences in mental healthcare and pharmaceutical costs.

**Fig. 1:**
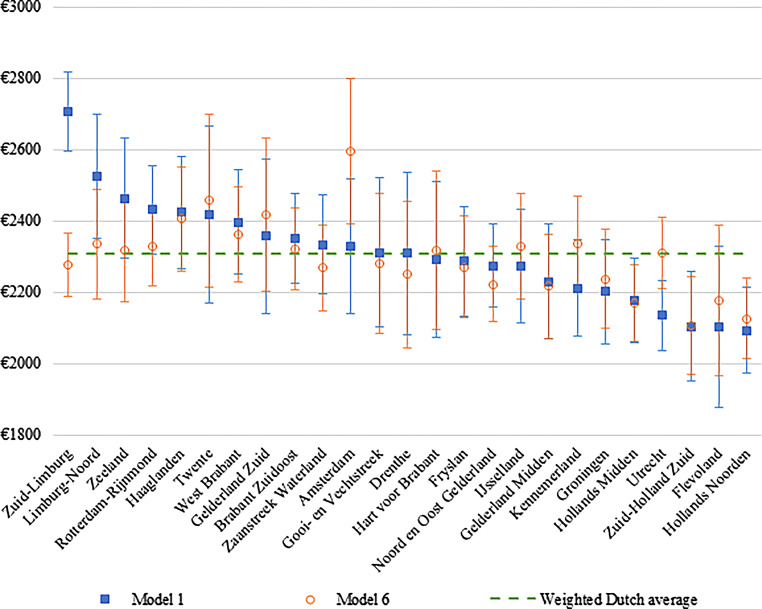
Marginal costs of region for total healthcare costs based on unadjusted costs (*Model* *1*) and fully adjusted costs (*Model* *6*). (Model 1 was only adjusted for region. In Model 6, the association between region and healthcare costs was adjusted for demographic factors, socioeconomic status (*SES*), general and mental health, lifestyle, loneliness, and mastery. The marginal costs in Model 6 reflect the average costs per person in a specific region if the population of this region shared the same level of demographic factors, SES, general and mental health, lifestyle, loneliness, and mastery as the entire Dutch population. Registry data: age, gender, migration background and household income. Self-reported data: marital status, education, income inadequacy, self-rated health, chronic disease, psychological distress, lifestyle, loneliness, and mastery)

The marginal costs for total healthcare are shown in Fig. 1, with the greatest (positive) difference seen in Models 1 and 6 for Zuid-Limburg. Here, the average marginal costs decreased significantly from € 2,705 per person (based on uncorrected data) to € 2,277 per person (based on corrected data). The biggest (negative) difference was observed in the region of Amsterdam, where the average marginal costs rose from € 2,327 to € 2,595 per person after adjusting for demographic factors, SES, general and mental health, lifestyle, loneliness, and mastery. For the other cost categories, the confidence intervals for Zuid-Limburg did not overlap or only overlapped minimally. The average marginal costs decreased for GP consultations (from € 52 to € 48), pharmaceuticals (from € 318 to € 286), and specialized care (from € 1,453 to € 1,257) (see Appendix Figures A1–A4).

## Discussion

We observed large regional differences in healthcare costs in the Netherlands. Based on total healthcare costs, regional differences could be explained. In addition to the most common factors (demographic factors and SES), health status, lifestyle, loneliness, and mastery contributed to these variations in different ways. General and mental health explained a large part of the regional differences in healthcare costs. Lifestyle, loneliness, and mastery contributed directly and to a small extent to the explanation of regional differences in healthcare costs. Our other published study showed that lifestyle, loneliness, and mastery contributed to regional differences in health and thus indirectly to regional differences in healthcare costs as well [10]. This study presented regional differences based on the reference region of Zuid-Limburg. In the Dutch-language tool Compare Regions for Health and Healthcare Costs (Regiovergelijker gezondheid en zorgkosten), users can choose their own reference region and compare the results of the uncorrected model (Model 1) and the comprehensive adjusted model (Model 6) [19].

With regard to various healthcare cost categories, a number of findings stand out. First, residents of Zuid-Limburg incurred the highest GP consultation costs, even in the most comprehensive model. In other words, with the same demographic factors, SES, general and mental health, lifestyle, loneliness, and level of mastery, residents of Zuid-Limburg still had more GP consultations. This corresponds with earlier, albeit anecdotal, evidence from research into the health inequalities in Limburg [20]. Even though cost savings for the entire healthcare system are small (in 2017, GP consultation costs represented 1.7% of total healthcare costs), the higher demand for GP care does correspond with the increasing pressure that GPs experience, especially during the coronavirus pandemic [21]. GPs increasingly have to deal with patients with (psycho)social problems, who require other types of care [22, 23]. The results of this study suggest that by intervening in the (causes of) socioeconomic problems, differences in lifestyle, loneliness and mastery, the differences in GP costs—and thus the pressure on GPs—can perhaps be reduced.

Second, regional differences in mental healthcare costs (reimbursed under the Healthcare Insurance Act) were less frequently observed, but they were more persistent than regional differences in other healthcare cost categories. Even when we included all factors in this study, regional differences remained. Further research is needed to determine which factors can further contribute to explaining regional differences in mental healthcare costs. A further breakdown of mental healthcare costs (specialist versus generalist care, inpatient care versus outpatient care versus long-term mental healthcare) possibly provides more insight into regional cost differences. In addition, combining cost data from the Healthcare Insurance Act and the Long-Term Care Act offers opportunities for improved analyses of long-term, inpatient mental healthcare costs. Moreover, differences between urban and less urban areas within regions or between areas on a smaller geographic scale, such as at the municipal, district, or neighborhood level, may also contribute to explaining regional differences in healthcare costs in future research.

Our findings help health insurance companies and policymakers justify investments in basic conditions for health, lifestyle, loneliness, and mastery. Although the direct contribution of lifestyle, loneliness, and mastery seems limited, these contributions still result in large variations in healthcare costs between regions. The differences in GP consultation costs, for example, became significantly smaller in 23 regions, and the differences in mental healthcare costs were significantly smaller in three regions. At the population level (difference in marginal costs per person times the average number of residents aged 19 years and over, per region in 2017), we were able to explain € 1.6 million in GP consultation costs and € 4 million in mental healthcare costs (see Appendix Tables A8 and A9).

In addition to this direct contribution, the three factors also contributed to the explanation of regional health differences [10]. These health differences play a major role in explaining variations in regional healthcare costs. Given these results, the three factors appear to be related to healthcare costs both directly and indirectly. This offers leads for investments in prevention programs and facilities aimed at reducing unhealthy lifestyles and loneliness and improving mastery. In collaboration with partners in social work and informal care, these investments could lead to savings for the medical sector and health insurance companies.

A second implication is extension of the set of individual characteristics in determining healthcare budgets. For example, in order to prevent risk selection and premium differentiation, health insurance companies receive a contribution from the Health Insurance Fund in addition to the premiums collected. This contribution, the risk equalization, is calculated based on, among other things, demographic and socioeconomic characteristics and zip code area [24]. However, certain regions are faced with budget shortages. The results of this research show that factors such as lifestyle, loneliness, mastery, and health contribute to regional differences in healthcare costs. Data on these factors are not available for the entire population and can therefore not be included in the risk equalization calculations for now. As a result, health insurance companies cannot adequately prepare for possible shortages. However, shortages are not unique to health insurance companies, as they also arise in local government budgets with regard to the Social Support Act, Youth Care Act, and the Participation Act in, for example, Zuid-Limburg [25] and Zeeland [26]. The distribution models for these budgets also do not take into account all individual factors that contribute to health inequalities and healthcare costs [20].

A potential limitation of this study is the composition of the sample. Selection bias may occur as certain groups of people are less likely to participate in research, such as those with a lower SES and/or poorer health [27]. The sampling method and weighting factors were used to counter this limitation. In addition, the use of the Health Survey sample may have resulted in an underestimation of mental healthcare costs. Even though the size of the group of mental healthcare users in the sample was in line with the national average (approximately 5%), inpatient mental health patients were not included, while they incur (extremely) high costs. This underrepresentation did apply to every region in the Netherlands, making a regional comparison possible, with the assumption that there were no regional differences in policies on institutionalization or inpatient admission in mental healthcare. A second limitation is the use of cross-sectional data. As a result, we could only analyze associations and were unable to draw any causal conclusions. We know that an unhealthy lifestyle and loneliness are related to healthcare costs [6–9]. However, we do not know whether lifestyle and loneliness lead to poor health and thereby to higher healthcare costs, nor whether poor health leads to (more) loneliness and an unhealthy lifestyle. Little is yet known about the relationship between mastery and healthcare costs. Our results show that respondents with sufficient mastery have lower healthcare costs (not tabulated).

The association between mastery and healthcare costs is increasingly important as the role of the citizen (or patient) is becoming more important as society and the healthcare system are increasingly complex [28]. In addition, terms such as “mastery”, “self-management”, and “positive health” are conceptually different, while at the same time they overlap in practice [29]. The Pearlin Mastery Scale specifically relates to individual problem-solving skills. In self-management and positive health, emphasis is placed on an adaptive capacity to deal with challenges, using the individual’s own skills, in combination with their social network and professional support. One of the reasons why residents of Zuid-Limburg have the lowest mastery score may be related to the disappearance of the social welfare system that was set up by the Catholic Church and the mining companies in the past [10]. In the measurement of mastery, support by a social network and professionals is not explicitly included. Hypothetically, low mastery may also be related to inadequate social networks and/or professional help. Further research into the association between mastery and self-management and its relationship with healthcare costs is required.

In conclusion, the factors included in this study could largely explain regional differences in total healthcare costs. This offers leads for preventive investments aimed at a wide range of individual factors in conjunction. This does not imply an approach that is merely focused on combating an unhealthy lifestyle but also on combating loneliness and improving mastery. This calls for a comprehensive approach and further cooperation between the social and medical sector and *with* education, housing, and public spaces that may affect health skills and self-reliance. In addition, the most vulnerable regions benefit from reconsidering distribution models for risk equalization, since a wider range of individual factors contribute to health, and thus healthcare costs, than the factors that are currently taken into account.

## Supplementary Information

Appendix
