# Supplementary material for: Regional differences in healthcare costs further explained: The contribution of health, lifestyle, loneliness and mastery
Source: TSG. 2022 Nov 2;100(4):189–96. doi: 10.1007/s12508-022-00369-4 (PMC9628485; doi:10.1007/s12508-022-00369-4)
Supplement: Supplementary file 1 — Appendix [file 12508_2022_369_MOESM1_ESM.pdf]

Appendix

Table A1. Descriptive data (n = 334,721) based on unweighted data.

**Sample**		**N**	**(%)**
**Gender§**	Male	159,251	(47.6%)
	Female	175,470	(52.4%)
**Migration background§**	Dutch-born	294,573	(88.0%)
	Western migration background	28,204	(8.4%)
	Non-western migration background	11,944	(3.6%)
**Marital status***	Married/living together	224,234	(67.0%)
	Never married	35,899	(10.7%)
	Widowed	23,052	(6.9%)
	Divorced	31,356	(9.4%)
**Highest attained level of education***	Primary school	19,061	(5.7%)
	Lower vocational education	102,886	(30.7%)
	Middle vocational/secondary	106,341	(31.8%)
	Higher vocational/university	106,433	(31.8%)
**Standardized household income quartile§**	0-25%	42,250	(12.6%)
	26-50%	84,243	(25.2%)
	51-75%	98,107	(29.3%)
	76-100%	110,121	(32.9%)
**Self-perceived income inadequacy***	Inadequate. major concerns	9,490	(2.8%)
	Inadequate. some concerns	34,273	(10.2%)
	Adequate. minor concerns	115,299	(34.4%)
	Adequate. no concerns	175,659	(52.5%)
**Chronic disease***	None	203,330	(60.7%)
	At least one	131,391	(39.3%)
**Self-rated health***	Very (good)	247,707	(74.0%)
	Fair. (very) poor	87,104	(26.0%)
**Psychological distress***	No. low or moderate risk	319,533	(95.5%)
	High	15,188	(4.5%)
**Physical activity***	Insufficient	94,343	(28.2%)
	Sufficient	240,378	(71.8%)
**BMI***	Underweight (<18.5)	4,200	(1.3%)
	Normal (18.5-25)	152,321	(45.5%)
	Overweight (25-30)	128,977	(38.5%)
	Obese (30>)	49,223	(14.7%)
**Alcohol consumption***	Never	32,663	(9.8%)
	Moderate	275,392	(82.3%)
	Excessive	26,666	(8.0%)
**Smoking***	Never smoked	135,642	(40.5%)
	Former smoker	144,994	(43.3%)
	Current smoker	54,085	(16.2%)
		**Mean**	**(sd)**
**Age§**		59.2	(16.9)
**Loneliness***		2.8	(3.1)
**Mastery***		26.7	(5.2)

§Registry data. *Self-reported data (Health Survey 2016).

**Table A2. Missing data (n=457.150)**		
**Variable**	**N**	**(%)**
**Region***	0	(0)
**Age§**	0	(0)
**Gender§**	0	(0)
**Migration background§**	0	(0)
**Marital state***	11,192	(2.4)
**Highest attained level of education ***	31,422	(6.9)
**Standardized household income quartile§**	737	(0.2)
**Income inadequacy***	35,643	(7.8)
**Physical activity***	33,488	(7.3)
**BMI***	21,261	(4.7)
**Alcohol consumption***	36,967	(8.1)
**Smoking***	32,605	(7.1)
**Chronic disease***	8,808	(1.9)
**Self-rated health***	5,730	(1.3)
**Psychological distress***	20,103	(4.4)
**Loneliness***	36,364	(8.0)
**Mastery***	36,612	(8.0)

§Registry data. *Self-reported data (Health Survey 2016).

Table A3. Incidence Rate Ratios per region for total healthcare costs compared to Zuid-Limburg. Results from Poisson regressions (n= 334,721).

IRR (95%CI)	Model 1	Model 2	Model 3a	Model 3b	Model 3c
Zuid-Limburg	1.00 (ref)	1.00 (ref)	1.00 (ref)	1.00 (ref)	1.00 (ref)
Zuid-Holland-Zuid	0.78 (0.71-0.85)	0.82 (0.76-0.89)	0.91 (0.84-0.98)	0.89 (0.82-0.96)	0.84 (0.78-0.92)
Zeeland	0.91 (0.84-0.99)	0.90 (0.83-0.97)	0.94 (0.87-1.01)	0.97 (0.90-1.05)	0.92 (0.85-0.99)
Zaanstreek-Waterland	0.86 (0.80-0.93)	0.91 (0.85-0.98)	0.98 (0.92-1.05)	0.94 (0.88-1.00)	0.93 (0.87-0.99)
West-Brabant	0.89 (0.82-0.95)	0.92 (0.86-0.99)	0.96 (0.89-1.03)	1.01 (0.94-1.08)	0.94 (0.87-1.01)
Utrecht	0.79 (0.74-0.84)	0.92 (0.86-0.97)	0.99 (0.93-1.05)	0.96 (0.91-1.02)	0.94 (0.88-0.99)
Twente	0.89 (0.80-0.99)	0.96 (0.86-1.07)	1.06 (0.95-1.18)	1.00 (0.90-1.11)	0.97 (0.87-1.08)
Rotterdam-Rijnmond	0.90 (0.84-0.96)	0.94 (0.88-1.00)	1.01 (0.95-1.08)	0.97 (0.91-1.04)	0.96 (0.90-1.03)
Noord- en Oost-Gelderland	0.84 (0.79-0.90)	0.90 (0.84-0.95)	0.98 (0.92-1.05)	0.91 (0.85-0.97)	0.91 (0.85-0.97)
Limburg-Noord	0.93 (0.86-1.01)	0.95 (0.87-1.02)	0.99 (0.92-1.07)	0.98 (0.91-1.06)	0.97 (0.90-1.05)
Kennemerland	0.82 (0.76-0.88)	0.88 (0.82-0.95)	0.99 (0.92-1.06)	0.95 (0.88-1.01)	0.91 (0.85-.098)
IJsselland	0.84 (0.77-0.91)	0.95 (0.87-1.03)	1.04 (0.96-1.12)	0.95 (0.88-1.03)	0.97 (0.89-1.05)
Hollands Noorden	0.77 (0.72-0.83)	0.82 (0.77-0.88)	0.90 (0.84-0.96)	0.87 (0.81-0.93)	0.84 (0.79-0.90)
Holland-Midden	0.80 (0.75-0.86)	0.89 (0.83-0.95)	0.97 (0.91-1.04)	0.90 (0.84-0.96)	0.90 (0.84-0.96)
Hart voor Brabant	0.85 (0.76-0.94)	0.90 (0.81-1.00)	0.95 (0.86-1.05)	0.97 (0.88-1.08)	0.92 (0.83-1.02)
Haaglanden	0.90 (0.83-0.97)	0.98 (0.91-1.05)	1.05 (0.98-1.13)	0.99 (0.92-1.06)	0.98 (0.91-1.06)
Groningen	0.81 (0.75-0.88)	0.88 (0.82-0.96)	0.96 (0.89-1.04)	0.89 (0.83-0.96)	0.92 (0.85-0.99)
Gooi en Vechtstreek	0.85 (0.77-0.94)	0.89 (0.81-0.98)	0.98 (0.89-1.07)	0.96 (0.87-1.05)	0.92 (0.83-1.01)
Gelderland-Zuid	0.87 (0.79-0.96)	0.97 (0.88-1.07)	1.05 (0.95-1.06)	1.01 (0.92-1.12)	0.98 (0.89-1.08)
Gelderland-Midden	0.82 (0.76-0.90)	0.90 (0.83-0.97)	0.98 (0.90-1.06)	0.91 (0.84-0.99)	0.91 (0.84-0.98)
Friesland	0.85 (0.78-0.91)	0.87 (0.80-0.94)	0.98 (0.91-1.06)	0.91 (0.85-0.99)	0.89 (0.83-0.97)
Flevoland	0.78 (0.69-0.87)	0.89 (0.80-0.99)	0.95 (0.85-1.06)	0.90 (0.81-1.00)	0.90 (0.81-1.01)
Drenthe	0.85 (0.77-0.95)	0.86 (0.78-0.96)	0.96 (0.87-1.06)	0.92 (0.83-1.02)	0.89 (0.80-0.99)
Brabant-Zuidoost	0.87 (0.81-0.93)	0.92 (0.86-0.99)	0.95 (0.89-1.02)	1.00 (0.93-1.06)	0.94 (0.88-0.99)
Amsterdam	0.86 (0.79-0.94)	0.99 (0.91-1.09)	1.10 (1.01-1.21)	1.06 (0.97-1.15)	1.03 (0.94-1.12)

Tabel A3 continued.

IRR (95%CI)	Model 4	Model 5a	Model 5b	Model 5c	Model 6
Zuid-Limburg	1.00 (ref)	1.00 (ref)	1.00 (ref)	1.00 (ref)	1.00 (ref)
Zuid-Holland-Zuid	0.93 (0.86-1.01)	0.93 (0.86-1.01)	0.93 (0.86-1.01)	0.93 (0.86-1.00)	0.92 (0.86-0.99)
Zeeland	0.99 (0.92-1.07)	1.02 (0.95-1.10)	1.00 (0.93-1.07)	0.99 (0.92-1.06)	1.02 (0.95-1.09)
Zaanstreek-Waterland	0.98 (0.92-1.05)	1.00 (0.93-1.07)	0.98 (0.92-1.05)	0.99 (0.93-1.06)	1.00 (0.93-1.06)
West-Brabant	1.03 (0.96-1.10)	1.04 (0.97-1.12)	1.03 (0.96-1.10)	1.02 (0.95-1.10)	1.04 (0.97-1.11)
Utrecht	1.00 (0.95-1.06)	1.02 (0.96-1.08)	1.00 (0.94-1.06)	1.01 (0.95-1.07)	1.01 (0.96-1.08)
Twente	1.06 (0.95-1.18)	1.09 (0.98-1.21)	1.06 (0.95-1.17)	1.06 (0.96-1.18)	1.08 (0.97-1.20)
Rotterdam-Rijnmond	1.02 (0.96-1.08)	1.02 (0.96-1.09)	1.02 (0.95-1.08)	1.02 (0.96-1.09)	1.02 (0.96-1.09)
Noord- en Oost-Gelderland	0.97 (0.91-1.03)	0.99 (0.93-1.05)	0.96 (0.91-1.03)	0.97 (0.91-1.03)	0.98 (0.92-1.04)
Limburg-Noord	1.01 (0.93-1.09)	1.02 (0.95-1.11)	1.01 (0.93-1.09)	1.01 (0.94-1.09)	1.03 (0.95-1.11)
Kennemerland	1.01 (0.94-1.08)	1.03 (0.96-1.10)	1.00 (0.94-1.08)	1.02 (0.95-1.09)	1.03 (0.96-1.10)
IJsselland	1.02 (0.94-1.10)	1.04 (0.96-1.12)	1.01 (0.94-1.09)	1.01 (0.94-1.09)	1.02 (0.95-1.10)
Hollands Noorden	0.91 (0.85-0.97)	0.93 (0.87-0.99)	0.91 (0.85-0.97)	0.92 (0.86-0.98)	0.93 (0.87-0.99)
Holland-Midden	0.96 (0.90-1.02)	0.98 (0.92-1.04)	0.95 (0.89-1.02)	0.94 (0.88-1.00)	0.95 (0.89-1.01)
Hart voor Brabant	1.00 (0.90-1.11)	1.02 (0.92-1.13)	1.00 (0.90-1.10)	1.00 (0.90-1.11)	1.02 (0.92-1.13)
Haaglanden	1.04 (0.96-1.12)	1.05 (0.98-1.13)	1.03 (0.96-1.11)	1.05 (0.97-1.13)	1.06 (0.98-1.14)
Groningen	0.96 (0.89-1.04)	0.98 (0.91-1.05)	0.96 (0.89-1.03)	0.97 (0.90-1.05)	0.98 (0.91-1.06)
Gooi en Vechtstreek	1.00 (0.91-1.10)	1.00 (0.91-1.10)	1.00 (0.91-1.09)	1.01 (0.92-1.11)	1.00 (0.91-1.10)
Gelderland-Zuid	1.06 (0.96-1.17)	1.07 (0.97-1.18)	1.06 (0.96-1.16)	1.06 (0.96-1.17)	1.06 (0.96-1.17)
Gelderland-Midden	0.97 (0.89-1.04)	0.98 (0.91-1.06)	0.96 (0.89-1.03)	0.97 (0.89-1.04)	0.97 (0.90-1.05)
Friesland	0.99 (0.91-1.06)	1.00 (0.93-1.08)	0.98 (0.91-1.06)	0.99 (0.92-1.06)	1.00 (0.93-1.07)
Flevoland	0.94 (0.85-1.05)	0.95 (0.85-1.05)	0.94 (0.85-1.04)	0.95 (0.85-1.06)	0.96 (0.86-1.06)
Drenthe	0.98 (0.89-1.08)	0.99 (0.89-1.09)	0.98 (0.88-1.08)	0.99 (0.89-1.09)	0.99 (0.89-1.09)
Brabant-Zuidoost	1.01 (0.95-1.17)	1.03 (0.97-1.10)	1.01 (0.94-1.07)	1.00 (0.94-1.07)	1.02 (0.96-1.09)
Amsterdam	1.12 (1.03-1.22)	1.14 (1.04-1.25)	1.12 (1.02-1.22)	1.13 (1.04-1.24)	1.14 (1.04-1.25)

IRR: Incidence Rate Ratio. CI: confidence interval. Model 1: region. Model 2: region, demographic factors, and SES. Model 3a: region, demographic factors, SES, and self-rated health. Model 3b: region, demographic factors, SES and chronic disease. Model 3c: region, demographic factors, SES and psychological distress. Model 4: region, demographic factors, SES, self-rated health, chronic disease, and psychological distress. Model 5a: region, demographic factors, SES, self-rated health, chronic disease, psychological distress, and lifestyle. Model 5b: region, demographic factors, SES, self-rated health, chronic disease, psychological distress, and loneliness. Model 5c: region, demographic factors, SES, self-rated health, chronic disease, psychological distress, and mastery. Model 6: region, demographic factors, SES, self-rated health, chronic disease, psychological distress, lifestyle, loneliness, and mastery. Registry data: age, gender, migration background and household income. Self-reported data: marital status, education, income inadequacy, self-rated health, chronic disease, psychological distress, lifestyle, loneliness, and mastery.

Table A4. Incidence Rate Ratios per region for GP consult costs compared to Zuid-Limburg. Results from Zero-inflated negative binomial regressions (n= 334,721).

IRR (95%CI)	Model 1		Model 2		Model 3a		Model 3b		Model 3c	
		Infl.		Infl.		Infl.		Infl.		Infl.
Zuid-Limburg	1.00 (ref)	1.00 (ref)	1.00 (ref)	1.00 (ref)	1.00 (ref)	1.00 (ref)	1.00 (ref)	1.00 (ref)	1.00 (ref)	1.00 (ref)
Zuid-Holland-Zuid	0.82	1.14	0.85	1.13	0.89	1.10	0.87	1.10	0.87	1.12
	(0.79-0.86)	(1.02-1.28)	(0.82-0.89)	(1.01-1.27)	(0.85-0.92)	(0.98-1.23)	(0.84-0.91)	(0.98-1.23)	(0.83-0.91)	(1.00-1.25)
Zeeland	0.81	1.18	0.81	1.22	0.83	1.21	0.83	1.19	0.82	1.22
	(0.77-0.84)	(1.07-1.31)	(0.78-0.84)	(1.10-1.35)	(0.80-0.86)	(1.09-1.35)	(0.08-0.86)	(1.07-1.32)	(0.79-0.85)	(1.10-1.35)
Zaanstreek-	0.84	1.08	0.86	1.10	0.89	1.08	0.87	1.10	0.87	1.10
Waterland	(0.81-0.87)	(0.99-1.19)	(0.83-0.89)	(1.00-1.21)	(0.86-0.92)	(0.98-1.18)	(0.84-0.90)	(1.00-1.20)	(0.84-0.90)	(1.00-1.21)
West-Brabant	0.76	1.13	0.80	1.13	0.81	1.12	0.82	1.09	0.80	1.13
	(0.74-0.79)	(1.02-1.24)	(0.83-0.89)	(1.02-1.24)	(0.78-0.84)	(1.02-1.24)	(0.79-0.84)	(0.99-1.20)	(0.78-0.83)	(1.02-1.24)
Utrecht	0.79	1.29	0.85	1.21	0.87	1.18	0.86	1.19	0.86	1.20
	(0.77-0.81)	(1.20-1.39)	(0.83-0.88)	(1.12-1.30)	(0.85-0.90)	(1.01-1.28)	(0.84-0.89)	(1.10-1.28)	(0.84-0.88)	(1.12-1.30)
Twente	0.74	1.50	0.78	1.46	0.81	1.42	0.79	1.45	0.79	1.47
	(0.71-0.77)	(1.37-1.65)	(0.75-0.81)	(1.33-1.61)	(0.78-0.84)	(1.30-1.57)	(0.76-0.82)	(1.32-1.60)	(0.76-0.82)	(1.34-1.62)
Rotterdam-	0.87	2.11	0.87	2.21	0.90	2.18	0.88	2.22	0.88	2.20
Rijnmond	(0.84-0.90)	(1.97-2.27)	(0.85-0.90)	(2.05-2.38)	(0.87-0.92)	(2.03-2.35)	(0.86-0.91)	(2.06-2.40)	(0.86-0.91)	(2.05-2.37)
Noord- en Oost-	0.76	1.25	0.80	1.24	0.82	1.21	0.80	1.24	0.80	1.24
Gelderland	(0.74-0.79)	(1.14-1.36)	(0.77-0.82)	(1.14-1.35)	(0.80-0.85)	(1.11-1.31)	(0.77-0.82)	(1.14-1.36)	(0.77-0.82)	(1.14-1.35)
Limburg-Noord	0.88	1.04	0.89	1.05	0.92	1.04	0.91	1.04	0.90	1.05
	(0.85-0.91)	(0.96-1.14)	(0.87-0.92)	(0.96-1.15)	(0.89-0.94)	(0.95-1.13)	(0.88-0.93)	(0.95-1.14)	(0.88-0.93)	(0.96-1.15)
Kennemerland	0.79	1.08	0.83	1.07	0.87	1.04	0.85	1.05	0.84	1.07
	(0.76-0.82)	(0.99-1.18)	(0.81-0.86)	(0.98-1.17)	(0.84-0.90)	(0.95-1.14)	(0.82-0.88)	(0.96-1.15)	(0.81-0.87)	(0.97-1.17)
IJsselland	0.74	1.28	0.82	1.20	0.84	1.18	0.82	1.21	0.82	1.20
	(0.71-0.78)	(1.15-1.43)	(0.78-0.85)	(1.08-1.34)	(0.81-0.88)	(1.06-1.32)	(0.78-0.85)	(1.09-1.35)	(0.79-0.86)	(1.08-1.34)
Hollands Noorden	0.78	1.25	0.80	1.25	0.83	1.22	0.81	1.22	0.81	1.25
	(0.75-0.81)	(1.14-1.36)	(0.77-0.83)	(1.15-1.36)	(0.80-0.85)	(1.12-1.33)	(0.79-0.84)	(1.12-1.34)	(0.78-0.84)	(1.14-1.36)
Holland-Midden	0.79	1.22	0.83	1.17	0.91	1.14	0.83	1.18	0.84	1.17
	(0.77-0.82)	(1.12-1.32)	(0.81-0.86)	(1.08-1.27)	(0.88-0.94)	(1.05-1.23)	(0.81-0.86)	(1.09-1.28)	(0.81-0.86)	(1.08-1.27)
Hart voor Brabant	0.85	1.10	0.89	1.07	0.88	1.06	0.91	1.04	0.89	1.07
	(0.82-0.88)	(1.01-1.19)	(0.86-0.91)	(0.98-1.16)	(0.85-0.91)	(0.97-1.15)	(0.88-0.94)	(0.96-1.13)	(0.87-0.92)	(0.98-1.16)
Haaglanden	0.87	1.11	0.85	1.12	0.90	1.12	0.86	1.12	0.85	1.12
	(0.83-0.90)	(1.11-1.13)	(0.82-0.89)	(1.11-1.13)	(0.87-0.93)	(1.11-1.13)	(0.83-0.89)	(1.12-1.13)	(0.82-0.88)	(1.11-1.13)
Groningen	0.85	1.38	0.88	1.31	0.90	1.28	0.88	1.32	0.90	1.29
	(0.82-0.88)	(1.26-1.52)	(0.85-0.92)	(1.19-1.43)	(0.86-0.94)	(1.17-1.40)	(0.85-0.92)	(1.20-1.45)	(0.86-0.93)	(1.18-1.42)
Gooi en	0.84	1.10	0.88	1.10	0.84	1.07	0.90	1.07	0.88	1.10
Vechtstreek	(0.81-0.88)	(0.97-1.25)	0.84-0.92)	(0.97-1.25)	(0.81-0.88)	(0.95-1.21)	(0.86-0.94)	(0.95-1.21)	(0.84-0.92)	(0.97-1.24)
Gelderland-Zuid	0.85	1.25	0.89	1.19	0.91	1.16	0.90	1.17	0.89	1.19
	(0.82-0.88)	(1.14-1.37)	(0.86-0.92)	(1.08-1.30)	(0.88-0.94)	(1.06-1.27)	(0.87-0.93)	(1.07-1.28)	(0.86-0.92)	(1.09-1.30)

Gelderland-Midden	0.83	1.33	0.86	1.29	0.88	1.26	0.86	1.29	0.86	1.29
	(0.79-0.87)	(1.191-.48)	(0.83-0.90)	(1.16-1.44)	(0.85-0.92)	(1.13-1.40)	(0.83-0.90)	(1.16-1.44)	(0.83-0.90)	(1.16-1.44)
Friesland	0.80	1.31	0.81	1.32	0.84	1.28	0.82	1.30	0.82	1.31
	(0.77-0.83)	(1.20-1.44)	(0.79-0.84)	(1.21-1.44)	(0.82-0.87)	(1.17-1.40)	(0.80-0.85)	(1.19-1.42)	(0.79-0.85)	(1.20-1.43)
Flevoland	0.84	0.98	0.92	0.93	0.95	0.91	0.92	0.93	0.93	0.93
	(0.78-0.91)	(0.80-1.19)	(0.86-0.99)	(0.76-1.15)	(0.89-1.02)	(0.75-1.12)	(0.86-0.99)	(0.76-1.15)	(0.86-0.99)	(0.76-1.15)
Drenthe	0.83	1.11	0.85	1.14	0.88	1.10	0.87	1.12	0.86	1.13
	(0.79-0.87)	(0.98-1.25)	(0.81-0.89)	(1.00-1.29)	(0.85-0.93)	(0.97-1.25)	(0.83-0.91)	(0.99-1.26)	(0.82-0.90)	(1.00-1.28)
Brabant-Zuidoost	0.79	1.21	0.82	1.18	0.84	1.18	0.84	1.15	0.83	1.18
	(0.77-0.82)	(1.11-1.32)	(0.80-0.85)	(1.08-1.28)	(0.82-0.87)	(1.09-1.29)	(0.82-0.87)	(1.06-1.25)	(0.81-0.86)	(1.09-1.29)
Amsterdam	0.79	1.30	0.83	1.24	0.87	1.19	0.86	1.21	0.85	1.22
	(0.76-0.82)	(1.18-1.43)	(0.80-0.86)	(1.12-1.36)	(0.84-0.90)	(1.08-1.32)	(0.82-0.89)	(1.10-1.33)	(0.82-0.88)	(1.11-1.35)

Table A4 continued.

IRR	Model 4		Model 5a		Model 5b		Model 5c		Model 6	
IRR (95%CI)		Infl.		Infl.		Infl.		Infl.		Infl.
Zuid-Limburg	1.00 (ref)	1.00 (ref)	1.00 (ref)	1.00 (ref)	1.00 (ref)	1.00 (ref)	1.00 (ref)	1.00 (ref)	1.00 (ref)	1.00 (ref)
Zuid-Holland-Zuid	0.90	1.08	0.90	1.08	0.90	1.08	0.90	1.08	0.90	1.09
	(0.86-0.93)	(0.97-1.21)	(0.86-0.93)	(0.97-1.21)	(0.86-0.94)	(0.97-1.21)	(0.86-0.93)	(0.97-1.21)	(0.86-0.93)	(0.97-1.22)
Zeeland	0.84	1.19	0.84	1.19	0.84	1.19	0.83	1.20	0.84	1.20
	(0.81-0.87)	(1.07-1.32)	(0.81-0.87)	(1.08-1.33)	(0.81-0.87)	(1.07-1.32)	(0.80-0.86)	(1.08-1.33)	(0.80-0.87)	(1.08-1.33)
Zaanstreek-Waterland	0.89	1.08	0.89	1.09	0.90	1.08	0.89	1.08	0.90	1.09
	(0.86-0.92)	(0.99-1.19)	(0.87-0.92)	(0.99-1.19)	(0.87-0.93)	(0.99-1.19)	(0.87-0.92)	(0.99-1.19)	(0.87-0.93)	(0.99-1.19)
West-Brabant	0.82	1.10	0.82	1.10	0.82	1.10	0.82	1.10	0.82	1.10
	(0.79-0.85)	(0.99-1.21)	(0.79-0.85)	(1.00-1.21)	(0.79-0.85)	(0.99-1.21)	(0.79-0.85)	(1.00-1.21)	(0.79-0.85)	(1.00-1.21)
Utrecht	0.88	1.18	0.88	1.18	0.88	1.18	0.88	1.18	0.88	1.18
	(0.85-0.90)	(1.09-1.27)	(0.86-0.90)	(1.10-1.27)	(0.86-0.90)	(1.09-1.27)	(0.86-0.90)	(1.09-1.27)	(0.86-0.91)	(1.10-1.27)
Twente	0.81	1.44	0.81	1.45	0.81	1.44	0.81	1.44	0.81	1.46
	(0.78-0.84)	(1.31-1.59)	(0.79-0.84)	(1.32-1.60)	(0.78-0.84)	(1.31-1.59)	(0.78-0.84)	(1.31-1.59)	(0.79-0.84)	(1.32-1.60)
Rotterdam-Rijnmond	0.90	2.20	0.90	2.21	0.90	2.20	0.90	2.20	0.90	2.21
	(0.88-0.93)	(2.04-2.37)	(0.88-0.93)	(2.05-2.38)	(0.88-0.93)	(2.04-2.37)	(0.88-0.93)	(2.04-2.37)	(0.88-0.93)	(2.05-2.39)
Noord- en Oost-Gelderland	0.81	1.23	0.82	1.24	0.82	1.23	0.81	1.23	0.81	1.24
	(0.79-0.84)	(1.13-1.34)	(0.79-0.84)	(1.14-1.35)	(0.79-0.84)	(1.13-1.34)	(0.79-0.83)	(1.13-1.34)	(0.79-0.84)	(1.14-1.35)
Limburg-Noord	0.92	1.03	0.92	1.04	0.92	1.03	0.92	1.03	0.92	1.04
	(0.90-0.95)	(0.95-1.13)	(0.90-0.95)	(0.95-1.13)	(0.90-0.95)	(0.95-1.13)	(0.90-0.95)	(0.95-1.13)	(0.90-0.95)	(0.95-1.14)
Kennemerland	0.87	1.04	0.87	1.04	0.87	1.04	0.87	1.03	0.88	1.04
	(0.84-0.90)	(0.95-1.13)	(0.84-0.90)	(0.95-1.14)	(0.85-0.90)	(0.95-1.13)	(0.85-0.90)	(0.95-1.13)	(0.85-0.90)	(0.95-1.14)
IJsselland	0.83	1.20	0.84	1.21	0.84	1.20	0.83	1.20	0.84	1.22
	(0.80-0.87)	(1.08-1.34)	(0.80-0.87)	(1.09-1.35)	(0.80-0.87)	(1.08-1.34)	(0.80-0.87)	(1.08-1.34)	(0.80-0.87)	(1.09-1.36)
Hollands Noorden	0.83	1.21	0.83	1.22	0.83	1.21	0.84	1.21	0.84	1.22
	(0.81-0.86)	(1.11-1.33)	(0.81-0.86)	(1.12-1.33)	(0.81-0.86)	(1.11-1.33)	(0.81-0.86)	(1.11-1.32)	(0.81-0.87)	(1.12-1.33)
Holland-Midden	0.85	1.16	0.86	1.17	0.85	1.16	0.85	1.16	0.85	1.17
	(0.83-0.88)	(1.07-1.26)	(0.83-0.88)	(1.08-1.26)	(0.83-0.88)	(1.07-1.26)	(0.82-0.87)	(1.07-1.26)	(0.83-0.87)	(1.08-1.27)
Hart voor Brabant	0.92	1.04	0.92	1.05	0.92	1.04	0.92	1.04	0.92	1.05
	(0.89-0.94)	(0.96-1.13)	(0.89-0.95)	(0.96-1.14)	(0.89-0.95)	(0.96-1.13)	(0.89-0.94)	(0.96-1.13)	(0.90-0.95)	(0.97-1.14)
Haaglanden	0.87	1.12	0.87	1.12	0.87	1.12	0.87	1.12	0.87	1.12
	(0.84-0.90)	(0.84-0.90)	(0.84-0.90)	(1.12-1.13)	(0.84-0.91)	(1.12-1.13)	(0.84-0.90)	(1.11-1.13)	(0.84-0.90)	(1.12-1.13)
Groningen	0.90	1.30	0.90	1.31	0.91	1.30	0.91	1.30	0.91	1.31
	(0.87-0.94)	(1.18-1.42)	(0.87-0.94)	(1.19-1.44)	(0.87-0.94)	(1.18-1.42)	(0.87-0.94)	(1.18-1.42)	(0.87-0.94)	(1.19-1.44)
Gooi en Vechtstreek	0.91	1.06	0.91	1.06	0.91	1.06	0.91	1.06	0.92	1.06
	(0.87-0.95)	(0.94-1.20)	(0.87-0.95)	(0.94-1.20)	(0.87-0.95)	(0.94-1.20)	(0.87-0.95)	(0.93-1.20)	(0.88-0.96)	(0.94-1.20)
Gelderland-Zuid	0.91	1.16	0.91	1.16	0.91	1.16	0.91	1.16	0.91	1.17
	(0.88-0.94)	(1.06-1.27)	(0.88-0.94)	(1.06-1.27)	(0.88-0.94)	(1.06-1.27)	(0.88-0.94)	(1.06-1.27)	(0.88-0.94)	(1.07-1.27)

Gelderland-Midden	0.88 (0.84-0.91)	1.28 (1.15-1.43)	0.88 (0.85-0.91)	1.29 (1.15-1.44)	0.88 (0.84-0.91)	1.28 (1.15-1.43)	0.87 (0.84-0.91)	1.28 (1.15-1.43)	0.88 (0.84-0.91)	1.29 (1.16-1.44)
Friesland	0.84 (0.82-0.87)	1.28 (1.17-1.40)	0.85 (0.82-0.87)	1.28 (1.18-1.40)	0.85 (0.82-0.87)	1.28 (1.17-1.40)	0.84 (0.82-0.87)	1.28 (1.17-1.40)	0.85 (0.82-0.87)	1.29 (1.18-1.41)
Flevoland	0.95 (0.88-1.01)	0.92 (0.75-1.13)	0.95 (0.88-1.01)	0.92 (0.75-1.14)	0.95 (0.88-1.01)	0.92 (0.75-1.13)	0.95 (0.88-1.01)	0.92 (0.75-1.13)	0.95 (0.89-1.02)	0.93 (0.75-1.14)
Drenthe	0.89 (0.85-0.93)	1.10 (0.97-1.25)	0.89 (0.85-0.93)	1.11 (0.98-1.25)	0.89 (0.86-0.93)	1.10 (0.97-1.25)	0.90 (0.86-0.94)	1.10 (0.97-1.24)	0.90 (0.86-0.93)	1.11 (0.98-1.25)
Brabant-Zuidoost	0.85 (0.82-0.87)	1.16 (1.07-1.27)	0.85 (0.83-0.88)	1.17 (1.07-1.27)	0.85 (0.83-0.88)	1.16 (1.07-1.27)	0.85 (0.82-0.87)	1.17 (1.07-1.27)	0.85 (0.83-0.88)	1.17 (1.07-1.27)
Amsterdam	0.88 (0.85-0.91)	1.19 (1.08-1.31)	0.88 (0.85-0.91)	1.21 (1.10-1.33)	0.88 (0.85-0.92)	1.19 (1.08-1.31)	0.88 (0.85-0.91)	1.19 (1.08-1.31)	0.89 (0.86-0.92)	1.21 (1.10-1.33)

IRR: Incidence Rate Ratio. CI: confidence interval. Model 1: region. Model 2: region, demographic factors, and SES. Model 3a: region, demographic factors, SES, and self-rated health. Model 3b: region, demographic factors, SES and chronic disease. Model 3c: region, demographic factors, SES and psychological distress. Model 4: region, demographic factors, SES, self-rated health, chronic disease, and psychological distress. Model 5a: region, demographic factors, SES, self-rated health, chronic disease, psychological distress, and lifestyle. Model 5b: region, demographic factors, SES, self-rated health, chronic disease, psychological distress, and loneliness. Model 5c: region, demographic factors, SES, self-rated health, chronic disease, psychological distress, and mastery. Model 6: region, demographic factors, SES, self-rated health, chronic disease, psychological distress, lifestyle, loneliness, and mastery. Registry data: age, gender, migration background and household income. Self-reported data: marital status, education, income inadequacy, self-rated health, chronic disease, psychological distress, lifestyle, loneliness, and mastery.

Table A5. Incidence Rate Ratios per region for mental healthcare costs compared to Zuid-Limburg. Results from Zero-inflated negative binomial regressions (n= 334,721).

IRR (95%CI)	Model 1		Model 2		Model 3a		Model 3b		Model 3c	
		Infl.		Infl.		Infl.		Infl.		Infl.
Zuid-Limburg	1.00 (ref)	1.00 (ref)	1.00 (ref)	1.00 (ref)	1.00 (ref)	1.00 (ref)	1.00 (ref)	1.00 (ref)	1.00 (ref)	1.00 (ref)
Zuid-Holland-Zuid	0.47	1.36	0.54	1.22	0.54	1.15	0.57	1.18	0.51	1.14
	(0.36-0.62)	(1.09-1.68)	(0.41-0.70)	(0.98-1.53)	(0.42-0.70)	(0.92-1.44)	(0.42-0.76)	(0.94-1.47)	(0.40-0.65)	(0.91-1.42)
Zeeland	0.90	1.20	0.92	1.02	0.92	1.01	0.90	0.99	0.85	1.00
	(0.64-1.28)	(0.98-1.46)	(0.68-1.26)	(0.83-1.24)	(0.68-1.24)	(0.82-1.24)	(0.67-1.21)	(0.81-1.22)	(0.64-1.13)	(0.81-1.22)
Zaanstreek-	0.73	1.19	0.75	1.10	0.76	1.04	0.74	1.09	0.71	1.06
Waterland	(0.57-0.95)	(1.02-1.40)	(0.60-0.94)	(0.93-1.29)	(0.61-0.95)	(0.89-1.23)	(0.60-0.91)	(0.93-1.28)	(0.57-0.89)	(0.90-1.25)
West-Brabant	1.15	1.44	1.12	1.25	1.14	1.25	1.14	1.19	1.05	1.24
	(0.84-1.58)	(1.20-1.72)	(0.86-1.45)	(1.04-1.50)	(0.87-1.49)	(1.04-1.50)	(0.88-1.46)	(0.99-1.43)	(0.80-1.39)	(1.03-1.49)
Utrecht	0.78	0.99	0.84	0.96	0.84	0.91	0.86	0.93	0.83	0.90
	(0.64-0.95)	(0.88-1.12)	(0.70-1.00)	(0.84-1.09)	(0.71-1.00)	(0.80-1.04)	(0.72-1.02)	(0.82-1.06)	(0.69-1.00)	(0.79-1.03)
Twente	0.73	1.24	0.70	1.17	0.71	1.12	0.71	1.17	0.72	1.24
	(0.55-0.96)	(1.00-1.54)	(0.54-0.91)	(0.94-1.44)	(0.56-0.89)	(0.90-1.39)	(0.56-0.90)	(0.95-1.45)	(0.55-0.94)	(1.01-1.53)
Rotterdam-	0.91	1.15	0.89	1.28	0.94	1.23	0.90	1.25	0.90	1.22
Rijnmond	(0.69-1.19)	(1.01-1.32)	(0.72-1.11)	(1.11-1.47)	(0.75-1.18)	(1.07-1.42)	(0.73-1.11)	(1.08-1.43)	(0.71-1.15)	(1.06-1.41)
Noord- en Oost-	0.86	1.49	0.93	1.24	0.91	1.19	0.96	1.28	0.93	1.26
Gelderland	(0.66-1.12)	(1.27-1.74)	(0.73-1.18)	(1.06-1.47)	(0.71-1.15)	(1.01-1.40)	(0.75-1.22)	(1.08-1.51)	(0.72-1.21)	(1.06-1.49)
Limburg-Noord	0.90	1.43	0.99	1.23	1.01	1.20	1.03	1.22	1.03	1.17
	(0.66-1.22)	(1.23-1.67)	(0.74-1.34)	(1.06-1.44)	(0.73-1.40)	(1.02-1.40)	(0.75-1.42)	(1.04-1.43)	(0.74-1.43)	(0.99-1.37)
Kennemerland	0.78	1.09	0.80	1.01	0.80	0.94	0.81	0.97	0.77	0.96
	(0.55-1.11)	(0.93-1.28)	(0.62-1.03)	(0.86-1.18)	(0.64-1.01)	(0.80-1.11)	(0.64-1.01)	(0.82-1.14)	(0.61-0.97)	(0.81-1.13)
IJsselland	0.76	1.19	0.79	1.06	0.77	1.03	0.78	1.08	0.76	1.05
	(0.50-1.14)	(0.96-1.49)	(0.54-1.16)	(0.85-1.33)	(0.56-1.07)	(0.83-1.29)	(0.55-1.09)	(0.87-1.35)	(0.55-1.04)	(0.84-1.31)
Hollands Noorden	0.67	1.16	0.71	1.07	0.70	1.04	0.72	1.04	0.68	1.04
	(0.52-0.86)	(0.99-1.35)	(0.56-0.90)	(0.92-1.26)	(0.56-0.87)	(0.89-1.22)	(0.57-0.91)	(0.88-1.21)	(0.54-0.86)	(0.89-1.22)
Holland-Midden	0.62	1.13	0.68	1.03	0.68	0.98	0.69	1.05	0.64	1.03
	(0.50-0.77)	(0.98-1.30)	(0.55-0.82)	(0.89-1.19)	(0.56-0.81)	(0.85-1.14)	(0.57-0.83)	(0.91-1.22)	(0.52-0.78)	(0.89-1.20)
Hart voor Brabant	0.83	1.39	0.89	1.24	0.91	1.23	0.93	1.21	0.81	1.24
	(0.58-1.17)	(1.20-1.61)	(0.65-1.23)	(1.07-1.44)	(0.67-1.25)	(1.05-1.43)	(0.68-1.29)	(1.03-1.41)	(0.61-1.07)	(1.06-1.45)
Haaglanden	0.75	0.86	0.77	0.94	0.82	0.90	0.78	0.95	0.77	0.96
	(0.59-0.96)	(0.74-1.00)	(0.62-0.96)	(0.81-1.09)	(0.66-1.02)	(0.78-1.05)	(0.63-0.96)	(0.81-1.10)	(0.62-0.97)	(0.82-1.12)
Groningen	0.80	0.99	0.83	1.09	0.86	1.04	0.83	1.11	0.83	0.99
	(0.63-1.03)	(0.84-1.17)	(0.66-1.03)	(0.92-1.30)	(0.68-1.08)	(0.88-1.24)	(0.67-1.03)	(0.93-1.32)	(0.66-1.05)	(0.83-1.17)
Gooi en	0.70	1.23	0.74	1.11	0.74	1.06	0.77	1.08	0.70	1.08
Vechtstreek	(0.53-0.92)	(1.01-1.50)	(0.58-0.93)	(0.91-1.35)	(0.59-0.93)	(0.87-1.30)	(0.62-0.96)	(0.88-1.33)	(0.56-0.89)	(0.87-1.32)
Gelderland-Zuid	0.94	1.02	0.96	1.00	0.96	0.95	0.95	0.98	0.93	1.00
	(0.60-1.46)	(0.87-1.19)	(0.67-1.39)	(0.86-1.17)	(0.70-1.32)	(0.81-1.11)	(0.69-1.31)	(0.83-1.15)	(0.68-1.27)	(0.85-1.18)

Gelderland-Midden	1.04 (0.73-1.48)	1.25 (1.02-1.52)	1.17 (0.78-1.76)	1.19 (0.97-1.45)	1.16 (0.81-1.67)	1.16 (0.94-1.42)	1.14 (0.80-1.64)	1.22 (0.99-1.49)	1.15 (0.72-1.84)	1.25 (1.01-1.55)
Friesland	0.74 (0.58-0.95)	1.29 (1.09-1.52)	0.78 (0.62-0.99)	1.21 (1.02-1.43)	0.79 (0.62-0.99)	1.13 (0.96-1.34)	0.77 (0.62-0.95)	1.19 (1.00-1.41)	0.76 (0.60-0.97)	1.15 (0.97-1.37)
Flevoland	0.71 (0.48-1.04)	1.42 (1.03-1.97)	0.77 (0.54-1.09)	1.40 (1.00-1.98)	0.79 (0.57-1.10)	1.37 (0.96-1.97)	0.78 (0.56-1.10)	1.45 (1.02-2.08)	0.74 (0.53-1.03)	1.46 (1.00-2.11)
Drenthe	0.95 (0.61-1.48)	1.20 (0.94-1.52)	0.93 (0.65-1.32)	1.09 (0.86-1.39)	0.93 (0.67-1.29)	1.03 (0.80-1.31)	0.94 (0.68-1.30)	1.05 (0.82-1.34)	0.90 (0.66-1.24)	1.03 (0.81-1.32)
Brabant-Zuidoost	0.89 (0.71-1.11)	1.44 (1.22-1.70)	0.98 (0.78-1.23)	1.28 (1.08-1.51)	1.03 (0.82-1.29)	1.31 (1.10-1.55)	1.05 (0.84-1.31)	1.24 (1.04-1.47)	0.98 (0.78-1.22)	1.32 (1.11-1.57)
Amsterdam	0.81 (0.64-1.02)	0.71 (0.61-0.83)	0.86 (0.70-1.07)	0.86 (0.73-1.00)	0.88 (0.72-1.08)	0.77 (0.65-0.90)	0.91 (0.74-1.11)	0.80 (0.68-0.94)	0.88 (0.71-1.09)	0.78 (0.66-0.92)

Table A5 continued.

IRR (95%CI)	Model 4		Model 5a		Model 5b		Model 5c		Model 6	
		Infl.		Infl.		Infl.		Infl.		Infl.
Zuid-Limburg	1.00 (ref)	1.00 (ref)	1.00 (ref)	1.00 (ref)	1.00 (ref)	1.00 (ref)	1.00 (ref)	1.00 (ref)	1.00 (ref)	1.00 (ref)
Zuid-Holland-Zuid	0.54	1.10	0.53	1.11	0.54	1.08	0.54	1.11	0.53	1.10
	(0.42-0.69)	(0.88-1.38)	(0.42-0.68)	(0.88-1.39)	(0.42-0.68)	(0.86-1.36)	(0.42-0.69)	(0.88-1.40)	(0.42-0.68)	(0.88-1.39)
Zeeland	0.86	0.96	0.86	0.96	0.85	0.98	0.85	0.99	0.85	1.00
	(0.65-1.14)	(0.79-1.18)	(0.65-1.12)	(0.78-1.18)	(0.64-1.13)	(0.80-1.21)	(0.64-1.13)	(0.80-1.22)	(0.64-1.12)	(0.81-1.24)
Zaanstreek-	0.73	1.04	0.72	1.05	0.74	1.01	0.73	1.03	0.73	1.02
Waterland	(0.59-0.90)	(0.88-1.23)	(0.58-0.88)	(0.89-1.24)	(0.59-0.91)	(0.85-1.19)	(0.59-0.90)	(0.87-1.22)	(0.59-0.90)	(0.86-1.21)
West-Brabant	1.09	1.20	1.11	1.19	1.07	1.19	1.10	1.19	1.10	1.19
	(0.84-1.43)	(0.99-1.44)	(0.85-1.45)	(0.99-1.44)	(0.83-1.39)	(0.99-1.43)	(0.84-1.44)	(0.99-1.43)	(0.85-1.43)	(0.99-1.43)
Utrecht	0.85	0.87	0.85	0.88	0.86	0.85	0.86	0.86	0.86	0.85
	(0.71-1.02)	(0.76-0.99)	(0.71-1.01)	(0.77-1.00)	(0.72-1.03)	(0.74-0.97)	(0.72-1.03)	(0.75-0.98)	(0.72-1.03)	(0.75-0.98)
Twente	0.72	1.19	0.73	1.20	0.73	1.15	0.72	1.17	0.73	1.16
	(0.57-0.92)	(0.96-1.48)	(0.57-0.93)	(0.97-1.48)	(0.57-0.93)	(0.93-1.43)	(0.57-0.92)	(0.94-1.45)	(0.57-0.94)	(0.93-1.43)
Rotterdam-	0.93	1.20	0.93	1.20	0.93	1.17	0.94	1.17	0.93	1.16
Rijnmond	(0.74-1.17)	(1.04-1.38)	(0.74-1.16)	(1.04-1.39)	(0.75-1.17)	(1.01-1.35)	(0.75-1.18)	(1.02-1.36)	(0.75-1.16)	(1.01-1.34)
Noord- en Oost-	0.94	1.25	0.95	1.25	0.96	1.22	0.96	1.25	0.97	1.24
Gelderland	(0.73-1.22)	(1.05-1.48)	(0.73-1.24)	(1.06-1.48)	(0.74-1.26)	(1.03-1.45)	(0.74-1.25)	(1.05-1.48)	(0.74-1.28)	(1.05-1.47)
Limburg-Noord	1.06	1.16	1.06	1.16	1.06	1.16	1.06	1.15	1.05	1.16
	(0.75-1.50)	(0.98-1.36)	(0.75-1.49)	(0.98-1.36)	(0.75-1.48)	(0.98-1.36)	(0.76-1.47)	(0.98-1.35)	(0.76-1.46)	(0.98-1.37)
Kennemerland	0.79	0.91	0.78	0.92	0.80	0.89	0.79	0.89	0.79	0.89
	(0.63-0.98)	(0.77-1.08)	(0.63-0.96)	(0.78-1.09)	(0.64-1.00)	(0.75-1.05)	(0.64-0.98)	(0.75-1.05)	(0.64-0.97)	(0.75-1.05)
IJsselland	0.76	1.04	0.74	1.06	0.78	1.01	0.76	1.06	0.76	1.05
	(0.57-1.02)	(0.84-1.30)	(0.57-0.97)	(0.85-1.32)	(0.58-1.05)	(0.81-1.26)	(0.56-1.02)	(0.85-1.33)	(0.58-0.99)	(0.84-1.32)
Hollands Noorden	0.69	1.01	0.69	1.02	0.70	0.99	0.70	1.00	0.70	0.99
	(0.56-0.86)	(0.86-1.19)	(0.55-0.85)	(0.87-1.20)	(0.56-0.86)	(0.84-1.16)	(0.57-0.88)	(0.85-1.17)	(0.56-0.87)	(0.84-1.17)
Holland-Midden	0.65	1.02	0.65	1.02	0.66	1.00	0.66	1.05	0.66	1.03
	(0.54-0.79)	(0.88-1.19)	(0.54-0.79)	(0.88-1.19)	(0.55-0.80)	(0.86-1.16)	(0.55-0.80)	(0.90-1.22)	(0.55-0.80)	(0.89-1.20)
Hart voor Brabant	0.87	1.19	0.86	1.19	0.88	1.17	0.84	1.18	0.86	1.17
	(0.65-1.15)	(1.02-1.39)	(0.65-1.13)	(1.02-1.40)	(0.65-1.19)	(1.00-1.37)	(0.64-1.11)	(1.00-1.38)	(0.65-1.13)	(1.00-1.38)
Haaglanden	0.80	0.94	0.79	0.94	0.79	0.93	0.79	0.93	0.78	0.93
	(0.64-0.99)	(0.80-1.10)	(0.64-0.98)	(0.80-1.11)	(0.64-0.98)	(0.79-1.09)	(0.64-0.98)	(0.79-1.09)	(0.63-0.97)	(0.79-1.09)
Groningen	0.85	0.98	0.85	0.99	0.85	0.95	0.86	0.96	0.85	0.95
	(0.68-1.07)	(0.83-1.17)	(0.68-1.07)	(0.83-1.18)	(0.68-1.06)	(0.80-1.13)	(0.69-1.07)	(0.80-1.14)	(0.68-1.07)	(0.80-1.13)
Gooi en	0.74	1.05	0.74	1.05	0.74	1.01	0.75	1.03	0.75	1.01
Vechtstreek	(0.60-0.91)	(0.85-1.29)	(0.60-0.92)	(0.85-1.30)	(0.60-0.91)	(0.81-1.24)	(0.60-0.93)	(0.83-1.28)	(0.60-0.92)	(0.81-1.25)
Gelderland-Zuid	0.94	0.96	0.91	0.96	0.93	0.93	0.95	0.96	0.91	0.95
	(0.71-1.25)	(0.81-1.13)	(0.72-1.16)	(0.81-1.14)	(0.71-1.22)	(0.79-1.10)	(0.71-1.27)	(0.81-1.13)	(0.72-1.15)	(0.80-1.12)

Gelderland-Midden	1.13 (0.76-1.70)	1.23 (1.00-1.53)	1.14 (0.75-1.73)	1.25 (1.01-1.55)	1.16 (0.76-1.77)	1.22 (0.98-1.52)	1.14 (0.75-1.74)	1.24 (1.00-1.54)	1.17 (0.75-1.81)	1.25 (1.00-1.55)
Friesland	0.76 (0.61-0.95)	1.11 (0.93-1.32)	0.77 (0.62-0.97)	1.12 (0.94-1.33)	0.76 (0.61-0.95)	1.09 (0.92-1.30)	0.76 (0.61-0.96)	1.09 (0.92-1.30)	0.77 (0.62-0.96)	1.09 (0.92-1.30)
Flevoland	0.78 (0.55-1.09)	1.48 (1.01-2.19)	0.78 (0.55-1.11)	1.48 (1.00-2.18)	0.78 (0.55-1.09)	1.47 (0.99-2.17)	0.79 (0.55-1.13)	1.45 (0.99-2.12)	0.79 (0.55-1.13)	1.44 (0.98-2.12)
Drenthe	0.93 (0.69-1.25)	0.98 (0.77-1.25)	0.91 (0.67-1.24)	0.99 (0.77-1.26)	0.93 (0.68-1.26)	0.96 (0.75-1.23)	0.94 (0.69-1.26)	0.95 (0.74-1.22)	0.92 (0.67-1.26)	0.95 (0.75-1.22)
Brabant-Zuidoost	1.04 (0.83-1.29)	1.28 (1.07-1.52)	1.05 (0.84-1.31)	1.28 (1.07-1.52)	1.05 (0.84-1.31)	1.27 (1.06-1.52)	1.05 (0.85-1.31)	1.29 (1.08-1.54)	1.07 (0.86-1.33)	1.30 (1.08-1.55)
Amsterdam	0.91 (0.75-1.12)	0.71 (0.60-0.85)	0.90 (0.74-1.10)	0.73 (0.62-0.86)	0.93 (0.76-1.14)	0.69 (0.58-0.81)	0.92 (0.76-1.13)	0.69 (0.59-0.82)	0.92 (0.75-1.12)	0.70 (0.59-0.83)

IRR: Incidence Rate Ratio. CI: confidence interval. Model 1: region. Model 2: region, demographic factors, and SES. Model 3a: region, demographic factors, SES, and self-rated health. Model 3b: region, demographic factors, SES and chronic disease. Model 3c: region, demographic factors, SES and psychological distress. Model 4: region, demographic factors, SES, self-rated health, chronic disease, and psychological distress. Model 5a: region, demographic factors, SES, self-rated health, chronic disease, psychological distress, and lifestyle. Model 5b: region, demographic factors, SES, self-rated health, chronic disease, psychological distress, and loneliness. Model 5c: region, demographic factors, SES, self-rated health, chronic disease, psychological distress, and mastery. Model 6: region, demographic factors, SES, self-rated health, chronic disease, psychological distress, lifestyle, loneliness, and mastery. Registry data: age, gender, migration background and household income. Self-reported data: marital status, education, income inadequacy, self-rated health, chronic disease, psychological distress, lifestyle, loneliness, and mastery.

Table A6. Incidence Rate Ratios per region for pharmaceutical costs compared to Zuid-Limburg. Results from Zero-inflated negative binomial regressions (n= 334,721).

IRR (95%CI)	Model 1		Model 2		Model 3a		Model 3b		Model 3c	
		Infl.		Infl.		Infl.		Infl.		Infl.
Zuid-Limburg	1.00 (ref)	1.00 (ref)	1.00 (ref)	1.00 (ref)	1.00 (ref)	1.00 (ref)	1.00 (ref)	1.00 (ref)	1.00 (ref)	1.00 (ref)
Zuid-Holland-Zuid	1.12	1.23	1.17	1.19	1.18	1.12	1.19	1.11	1.20	1.09
	(0.84-1.50)	(1.08-1.40)	(0.90-1.51)	(1.04-1.35)	(0.98-1.42)	(0.99-1.26)	(1.02-1.38)	(0.99-1.24)	(1.04-1.38)	(0.97-1.22)
Zeeland	1.05	1.09	1.11	1.17	1.15	1.16	1.31	1.13	1.30	1.13
	(0.87-1.28)	(0.97-1.24)	(0.88-1.40)	(1.03-1.33)	(0.87-1.51)	(1.03-1.31)	(0.95-1.80)	(1.00-1.26)	(0.93-1.83)	(1.01-1.27)
Zaanstreek-Waterland	0.98	1.30	1.17	1.33	1.29	1.29	1.17	1.32	1.22	1.30
	(0.87-1.11)	(1.18-1.44)	(0.97-1.40)	(1.20-1.48)	(1.03-1.63)	(1.16-1.42)	(0.95-1.44)	(1.20-1.45)	(0.98-1.51)	(1.18-1.43)
West-Brabant	1.03	1.16	1.21	1.15	1.20	1.14	1.20	1.07	1.18	1.08
	(0.90-1.17)	(1.04-1.29)	(0.99-1.48)	(1.03-1.29)	(0.97-1.48)	(1.03-1.27)	(1.07-1.33)	(0.96-1.18)	(1.05-1.34)	(0.98-1.19)
Utrecht	0.88	1.63	1.00	1.38	1.04	1.33	1.04	1.33	1.06	1.32
	(0.79-0.98)	(1.50-1.77)	(0.89-1.11)	(1.27-1.50)	(0.94-1.16)	(1.23-1.44)	(0.96-1.13)	(1.23-1.44)	(0.97-1.15)	(1.22-1.42)
Twente	0.83	1.35	0.88	1.26	0.95	1.22	0.96	1.25	0.98	1.24
	(0.75-0.93)	(1.21-1.50)	(0.78-1.00)	(1.12-1.41)	(0.86-1.06)	(1.10-1.35)	(0.88-1.04)	(1.13-1.39)	(0.90-1.06)	(1.12-1.38)
Rotterdam-Rijnmond	1.10	1.23	1.23	1.19	1.27	1.15	1.18	1.16	1.20	1.15
	(0.99-1.23)	(1.12-1.34)	(1.08-1.41)	(1.09-1.30)	(1.12-1.44)	(1.06-1.25)	(1.07-1.30)	(1.07-1.26)	(1.09-1.32)	(1.06-1.25)
Noord- en Oost-Gelderland	0.95	1.35	1.05	1.37	1.15	1.32	1.10	1.39	1.13	1.37
	(0.84-1.06)	(1.23-1.49)	(0.92-1.19)	(1.24-1.51)	(1.00-1.32)	(1.20-1.44)	(0.94-1.29)	(1.27-1.52)	(0.97-1.32)	(1.25-1.50)
Limburg-Noord	0.94	1.06	0.92	1.09	0.95	1.07	0.98	1.08	0.98	1.07
	(0.86-1.02)	(0.95-1.17)	(0.83-1.01)	(0.98-1.21)	(0.86-1.05)	(0.97-1.18)	(0.91-1.05)	(0.98-1.18)	(0.91-1.06)	(0.97-1.17)
Kennemerland	0.85	1.49	0.93	1.44	1.02	1.38	1.05	1.38	1.08	1.37
	(0.76-0.94)	(1.35-1.65)	(0.82-1.06)	(1.30-1.59)	(0.89-1.17)	(1.25-1.51)	(0.91-1.21)	(1.26-1.52)	(0.94-1.24)	(1.25-1.50)
IJsselland	0.88	1.56	1.03	1.39	1.03	1.35	1.00	1.42	1.00	1.40
	(0.75-1.04)	(1.39-1.75)	(0.81-1.29)	(1.23-1.57)	(0.89-1.19)	(1.21-1.51)	(0.87-1.14)	(1.27-1.58)	(0.89-1.12)	(1.26-1.56)
Hollands Noorden	1.02	1.70	1.22	1.74	1.14	1.65	1.19	1.64	1.11	1.62
	(0.88-1.19)	(1.55-1.87)	(0.98-1.53)	(1.58-1.92)	(0.94-1.39)	(1.51-1.81)	(0.92-1.54)	(1.50-1.80)	(0.94-1.32)	(1.48-1.77)
Holland-Midden	0.99	1.45	1.05	1.32	1.06	1.27	0.98	1.34	0.99	1.32
	(0.82-1.19)	(1.32-1.59)	(0.89-1.24)	(1.21-1.45)	(0.94-1.21)	(1.16-1.38)	(0.89-1.09)	(1.23-1.46)	(0.91-1.09)	(1.22-1.44)
Hart voor Brabant	0.97	1.34	1.06	1.28	1.03	1.26	1.08	1.22	1.06	1.22
	(0.84-1.12)	(1.22-1.47)	(0.89-1.25)	(1.16-1.40)	(0.90-1.18)	(1.15-1.37)	(0.97-1.19)	(1.12-1.33)	(0.96-1.16)	(1.12-1.33)
Haaglanden	0.95	1.26	1.06	1.16	1.13	1.13	1.01	1.16	1.04	1.16
	(0.85-1.06)	(1.13-1.39)	(0.93-1.21)	(1.04-1.28)	(0.99-1.28)	(1.02-1.24)	(0.93-1.10)	(1.06-1.28)	(0.96-1.13)	(1.05-1.27)
Groningen	0.91	1.53	1.00	1.37	1.05	1.32	0.98	1.39	1.00	1.38
	(0.82-1.00)	(1.38-1.70)	(0.86-1.16)	(1.23-1.52)	(0.93-1.18)	(1.20-1.46)	(0.89-1.08)	(1.26-1.53)	(0.92-1.10)	(1.25-1.52)
Gooi en Vechtstreek	1.20	1.45	1.41	1.46	1.19	1.36	1.17	1.32	1.12	1.30
	(0.81-1.76)	(1.26-1.66)	(0.81-2.47)	(1.27-1.67)	(0.88-1.63)	(1.20-1.53)	(0.84-1.62)	(1.17-1.49)	(0.86-1.45)	(1.15-1.47)
Gelderland-Zuid	0.99	1.53	1.11	1.38	1.14	1.33	1.07	1.33	1.09	1.32
	(0.88-1.11)	(1.39-1.69)	(0.96-1.28)	(1.25-1.53)	(1.00-1.30)	(1.20-1.46)	(0.97-1.18)	(1.21-1.46)	(0.99-1.20)	(1.20-1.45)

Gelderland-Midden	0.97	1.51	0.98	1.39	1.04	1.34	0.97	1.40	0.98	1.38
	(0.84-1.11)	(1.34-1.70)	(0.87-1.12)	(1.23-1.57)	(0.91-1.19)	(1.19-1.50)	(0.89-1.06)	(1.25-1.57)	(0.89-1.08)	(1.24-1.55)
Friesland	1.00	1.38	1.05	1.41	1.10	1.34	1.03	1.35	1.05	1.33
	(0.86-1.17)	(1.25-1.53)	(0.88-1.25)	(1.27-1.56)	(0.95-1.27)	(1.21-1.47)	(0.92-1.15)	(1.23-1.48)	(0.94-1.17)	1.21-1.46)
Flevoland	0.97	1.31	1.14	1.16	1.20	1.14	1.07	1.18	1.08	1.17
	(0.77-1.23)	(1.05-1.64)	(0.86-1.51)	(0.92-1.47)	(0.85-1.68)	(0.92-1.41)	(0.89-1.27)	(0.95-1.45)	(0.89-1.32)	(0.95-1.43)
Drenthe	0.90	1.21	0.97	1.27	1.03	1.21	1.00	1.23	1.03	1.21
	(0.75-1.08)	(1.05-1.39)	(0.76-1.23)	(1.09-1.47)	(0.87-1.22)	(1.05-1.39)	(0.87-1.15)	(1.08-1.40)	(0.91-1.17)	(1.06-1.38)
Brabant-Zuidoost	0.90	1.34	0.95	1.28	0.99	1.29	1.02	1.21	1.03	1.24
	(0.82-0.98)	(1.21-1.47)	(0.85-1.07)	(1.16-1.41)	(0.87-1.12)	(1.17-1.41)	(0.94-1.12)	(1.11-1.33)	(0.92-1.14)	(1.13-1.35)
Amsterdam	1.26	1.78	1.33	1.44	1.46	1.35	1.24	1.34	1.30	1.32
	(1.07-1.48)	(1.60-1.97)	(1.12-1.59)	(1.29-1.60)	(1.21-1.76)	(1.22-1.50)	(1.07-1.44)	(1.22-1.48)	(1.12-1.51)	(1.20-1.46)

Table A6 continued.

IRR (95%CI)	Model 4		Model 5a		Model 5b		Model 5c		Model 6	
		Infl.		Infl.		Infl.		Infl.		Infl.
Zuid-Limburg	1.00 (ref)	1.00 (ref)	1.00 (ref)	1.00 (ref)	1.00 (ref)	1.00 (ref)	1.00 (ref)	1.00 (ref)	1.00 (ref)	1.00 (ref)
Zuid-Holland-Zuid	1.20	1.09	1.20	1.09	1.20	1.09	1.19	1.09	1.19	1.10
	(1.04-1.38)	(0.97-1.22)	(1.04-1.38)	(0.97-1.23)	(1.04-1.38)	(0.97-1.23)	(1.04-1.35)	(0.97-1.22)	(1.04-1.36)	(0.98-1.23)
Zeeland	1.30	1.13	1.32	1.12	1.30	1.13	1.29	1.13	1.31	1.12
	(0.93-1.83)	(1.01-1.27)	(0.94-1.85)	(1.00-1.26)	(0.93-1.82)	(1.01-1.27)	(0.92-1.81)	(1.01-1.27)	(0.93-1.84)	(1.00-1.26)
Zaanstreek-	1.22	1.30	1.22	1.30	1.22	1.31	1.22	1.30	1.22	1.31
Waterland	(0.98-1.51)	(1.18-1.43)	(0.99-1.51)	(1.18-1.43)	(0.98-1.51)	(1.19-1.44)	(0.98-1.53)	(1.18-1.43)	(0.98-1.51)	(1.19-1.44)
West-Brabant	1.18	1.08	1.20	1.08	1.18	1.08	1.18	1.08	1.19	1.08
	(1.05-1.34)	(0.98-1.19)	(1.06-1.35)	(0.97-1.19)	(1.05-1.34)	(0.98-1.19)	(1.04-1.33)	(0.98-1.19)	(1.05-1.34)	(0.98-1.19)
Utrecht	1.06	1.32	1.07	1.31	1.06	1.32	1.05	1.32	1.06	1.32
	(0.97-1.15)	(1.22-1.42)	(0.98-1.16)	(1.21-1.41)	(0.97-1.15)	(1.23-1.43)	(0.97-1.14)	(1.22-1.42)	(0.97-1.15)	(1.22-1.42)
Twente	0.98	1.24	0.99	1.25	0.98	1.25	0.98	1.24	0.98	1.26
	(0.90-1.06)	(1.12-1.38)	(0.91-1.07)	(1.13-1.38)	(0.90-1.06)	(1.13-1.39)	(0.90-1.06)	(1.12-1.38)	(0.91-1.06)	(1.13-1.39)
Rotterdam-	1.20	1.15	1.21	1.16	1.20	1.16	1.20	1.15	1.20	1.16
Rijnmond	(1.09-1.32)	(1.06-1.25)	(1.09-1.33)	(1.07-1.26)	(1.09-1.32)	(1.06-1.25)	(1.09-1.32)	(1.06-1.25)	(1.09-1.32)	(1.07-1.26)
Noord- en Oost-	1.13	1.37	1.14	1.38	1.13	1.38	1.13	1.37	1.13	1.39
Gelderland	(0.97-1.32)	(1.25-1.50)	(0.98-1.33)	(1.26-1.51)	(0.97-1.32)	(1.26-1.51)	(0.97-1.31)	(1.25-1.50)	(0.97-1.31)	(1.27-1.51)
Limburg-Noord	0.98	1.07	0.99	1.07	0.98	1.07	0.98	1.07	0.99	1.06
	(0.91-1.06)	(0.97-1.17)	(0.92-1.07)	(0.97-1.17)	(0.91-1.06)	(0.97-1.17)	(0.91-1.06)	(0.97-1.17)	(0.91-1.07)	(0.97-1.17)
Kennemerland	1.08	1.37	1.09	1.37	1.08	1.37	1.08	1.37	1.08	1.37
	(0.94-1.24)	(1.25-1.50)	(0.95-1.25)	(1.24-1.50)	(0.94-1.24)	(1.25-1.51)	(0.94-1.23)	(1.25-1.50)	(0.95-1.24)	(1.25-1.51)
IJsselland	1.00	1.40	1.01	1.41	1.00	1.41	0.99	1.40	1.01	1.42
	(0.89-1.12)	(1.26-1.56)	(0.90-1.14)	(1.26-1.57)	(0.89-1.12)	(1.26-1.57)	(0.88-1.12)	(1.25-1.56)	(0.89-1.13)	(1.27-1.59)
Hollands Noorden	1.11	1.62	1.12	1.62	1.11	1.63	1.11	1.62	1.12	1.63
	(0.94-1.32)	(1.48-1.77)	(0.94-1.34)	(1.48-1.77)	(0.94-1.32)	(1.49-1.78)	(0.94-1.33)	(1.48-1.77)	(0.94-1.34)	(1.49-1.78)
Holland-Midden	0.99	1.32	1.00	1.33	0.99	1.33	0.98	1.32	0.99	1.33
	(0.91-1.09)	(1.22-1.44)	(0.92-1.10)	(1.22-1.44)	(0.91-1.09)	(1.22-1.44)	(0.90-1.07)	(1.21-1.44)	(0.90-1.08)	(1.23-1.45)
Hart voor Brabant	1.06	1.22	1.07	1.22	1.06	1.23	1.05	1.22	1.06	1.22
	(0.96-1.16)	(1.12-1.33)	(0.97-1.17)	(1.12-1.32)	(0.96-1.16)	(1.13-1.34)	(0.96-1.16)	(1.12-1.33)	(0.97-1.17)	(1.12-1.33)
Haaglanden	1.04	1.16	1.05	1.15	1.04	1.16	1.04	1.16	1.05	1.16
	(0.96-1.13)	(1.05-1.27)	(0.96-1.14)	(1.05-1.27)	(0.96-1.13)	(1.05-1.28)	(0.96-1.14)	(1.05-1.27)	(0.96-1.14)	(1.05-1.27)
Groningen	1.00	1.38	1.00	1.39	1.00	1.38	1.00	1.38	1.00	1.39
	(0.92-1.10)	(1.25-1.52)	(0.92-1.10)	(1.26-1.53)	(0.92-1.10)	(1.25-1.52)	(0.91-1.10)	(1.25-1.52)	(0.91-1.09)	(1.26-1.53)
Gooi en	1.12	1.30	1.14	1.28	1.12	1.31	1.14	1.30	1.15	1.29
Vechtstreek	(0.86-1.45)	(1.15-1.47)	(0.88-1.47)	(1.13-1.44)	(0.87-1.45)	(1.16-1.48)	(0.86-1.50)	(1.15-1.47)	(0.88-1.50)	(1.14-1.46)
Gelderland-Zuid	1.09	1.32	1.10	1.31	1.09	1.33	1.08	1.32	1.09	1.32
	(0.99-1.20)	(1.20-1.45)	(0.99-1.21)	(1.19-1.44)	(0.99-1.20)	(1.21-1.46)	(0.98-1.19)	(1.20-1.45)	(0.99-1.20)	(1.20-1.45)

Gelderland-Midden	0.98 (0.89-1.08)	1.38 (1.24-1.55)	0.99 (0.90-1.09)	1.39 (1.24-1.56)	0.98 (0.89-1.08)	1.39 (1.24-1.56)	0.98 (0.89-1.07)	1.38 (1.23-1.55)	0.98 (0.89-1.08)	1.39 (1.24-1.56)
Friesland	1.05 (0.94-1.17)	1.33 (1.21-1.46)	1.06 (0.95-1.18)	1.33 (1.21-1.46)	1.05 (0.94-1.17)	1.33 (1.21-1.47)	1.05 (0.94-1.17)	1.33 (1.21-1.46)	1.06 (0.94-1.18)	1.33 (1.21-1.47)
Flevoland	1.08 (0.89-1.32)	1.17 (0.95-1.43)	1.09 (0.89-1.33)	1.17 (0.95-1.44)	1.08 (0.89-1.32)	1.17 (0.95-1.44)	1.08 (0.89-1.31)	1.17 (0.95-1.43)	1.08 (0.89-1.32)	1.18 (0.95-1.45)
Drenthe	1.03 (0.91-1.17)	1.21 (1.06-1.38)	1.04 (0.91-1.18)	1.23 (1.08-1.40)	1.03 (0.91-1.17)	1.22 (1.07-1.39)	1.03 (0.91-1.17)	1.21 (1.06-1.38)	1.03 (0.91-1.17)	1.23 (1.08-1.41)
Brabant-Zuidoost	1.03 (0.92-1.14)	1.24 (1.13-1.35)	1.04 (0.94-1.16)	1.22 (1.12-1.34)	1.03 (0.93-1.14)	1.24 (1.13-1.35)	1.02 (0.92-1.14)	1.23 (1.13-1.35)	1.04 (0.93-1.16)	1.23 (1.12-1.34)
Amsterdam	1.30 (1.12-1.51)	1.32 (1.20-1.46)	1.32 (1.13-1.53)	1.33 (1.21-1.47)	1.30 (1.12-1.51)	1.33 (1.20-1.47)	1.30 (1.12-1.50)	1.32 (1.20-1.46)	1.31 (1.13-1.53)	1.34 (1.21-1.48)

IRR: Incidence Rate Ratio. CI: confidence interval. Model 1: region. Model 2: region, demographic factors, and SES. Model 3a: region, demographic factors, SES, and self-rated health. Model 3b: region, demographic factors, SES and chronic disease. Model 3c: region, demographic factors, SES and psychological distress. Model 4: region, demographic factors, SES, self-rated health, chronic disease, and psychological distress. Model 5a: region, demographic factors, SES, self-rated health, chronic disease, psychological distress, and lifestyle. Model 5b: region, demographic factors, SES, self-rated health, chronic disease, psychological distress, and loneliness. Model 5c: region, demographic factors, SES, self-rated health, chronic disease, psychological distress, and mastery. Model 6: region, demographic factors, SES, self-rated health, chronic disease, psychological distress, lifestyle, loneliness, and mastery. Registry data: age, gender, migration background and household income. Self-reported data: marital status, education, income inadequacy, self-rated health, chronic disease, psychological distress, lifestyle, loneliness, and mastery.

Table A7. Incidence Rate Ratios per region for specialized care costs compared to Zuid-Limburg. Results from Zero-inflated negative binomial regressions (n= 334,721).

IRR (95%CI)	Model 1		Model 2		Model 3a		Model 3b		Model 3c	
		Infl.		Infl.		Infl.		Infl.		Infl.
Zuid-Limburg	1.00 (ref)	1.00 (ref)	1.00 (ref)	1.00 (ref)	1.00 (ref)	1.00 (ref)	1.00 (ref)	1.00 (ref)	1.00 (ref)	1.00 (ref)
Zuid-Holland-Zuid	0.83	1.15	0.83	1.12	0.92	1.07	0.91	1.07	0.84	1.11
	(0.75-0.91)	(1.04-1.26)	(0.75-0.91)	(1.01-1.23)	(0.84-1.00)	(0.96-1.18)	(0.84-1.00)	(0.97-1.18)	(0.76-0.93)	(1.00-1.22)
Zeeland	0.88	1.01	0.87	1.09	0.92	1.08	0.95	1.04	0.88	1.09
	(0.80-0.97)	(0.92-1.10)	(0.79-0.96)	(0.98-1.20)	(0.83-1.01)	(0.98-1.20)	(0.86-1.04)	(0.94-1.15)	(0.80-0.97)	(0.98-1.20)
Zaanstreek-	0.97	1.08	1.00	1.08	1.03	1.04	1.02	1.08	1.00	1.08
Waterland	(0.88-1.07)	(1.00-1.17)	(0.91-1.10)	(0.99-1.18)	(0.95-1.12)	(0.96-1.13)	(0.94-1.10)	(0.99-1.17)	(0.91-1.10)	(0.99-1.17)
West-Brabant	0.88	1.02	0.92	1.02	0.93	1.01	0.95	0.95	0.92	1.02
	(0.81-0.97)	(0.94-1.11)	(0.83-1.01)	(0.93-1.12)	(0.85-1.02)	(0.93-1.11)	(0.87-1.02)	(0.87-1.04)	(0.83-1.02)	(0.93-1.12)
Utrecht	0.90	1.32	0.97	1.16	1.03	1.13	1.02	1.13	0.98	1.16
	(0.83-0.98)	(1.25-1.41)	(0.89-1.06)	(1.09-1.24)	(0.95-1.12)	(1.05-1.20)	(0.94-1.10)	(1.05-1.21)	(0.90-1.07)	(1.08-1.24)
Twente	1.02	1.13	1.05	1.05	1.07	1.01	1.03	1.04	1.07	1.06
	(0.86-1.20)	(1.04-1.22)	(0.89-1.25)	(0.96-1.15)	(0.96-1.20)	(0.92-1.11)	(0.92-1.17)	(0.94-1.14)	(0.90-1.26)	(0.97-1.16)
Rotterdam-	0.95	1.10	1.03	1.04	1.09	1.01	1.06	1.03	1.04	1.03
Rijnmond	(0.87-1.03)	(1.03-1.17)	(0.94-1.14)	(0.97-1.12)	(1.01-1.19)	(0.94-1.09)	(0.98-1.15)	(0.96-1.11)	(0.94-1.14)	(0.96-1.11)
Noord- en Oost-	0.94	1.19	0.95	1.22	1.01	1.17	0.96	1.24	0.96	1.22
Gelderland	(0.86-1.02)	(1.11-1.28)	(0.87-1.04)	(1.13-1.31)	(0.94-1.10)	(1.09-1.27)	(0.88-1.03)	(1.15-1.34)	(0.87-1.05)	(1.13-1.32)
Limburg-Noord	0.99	0.96	1.03	1.00	1.07	0.97	1.02	0.97	1.04	0.99
	(0.89-1.12)	(0.89-1.04)	(0.89-1.20)	(0.92-1.08)	(0.90-1.26)	(0.89-1.05)	(0.92-1.13)	(0.89-1.06)	(0.89-1.21)	(0.91-1.08)
Kennemerland	0.92	1.16	0.97	1.13	1.07	1.08	1.03	1.09	0.98	1.13
	(0.84-1.02)	(1.08-1.26)	(0.87-1.07)	(1.04-1.23)	(0.96-1.19)	(1.00-1.18)	(0.95-1.12)	(1.00-1.19)	(0.88-1.09)	(1.04-1.22)
IJsselland	1.02	1.40	1.08	1.27	1.16	1.24	1.07	1.31	1.09	1.27
	(0.92-1.14)	(1.28-1.53)	(0.97-1.20)	(1.15-1.40)	(1.05-1.29)	(1.13-1.37)	(0.97-1.17)	(1.18-1.44)	(0.97-1.21)	(1.15-1.40)
Hollands Noorden	0.88	1.31	0.89	1.35	0.92	1.31	0.92	1.31	0.90	1.34
	(0.80-0.96)	(1.22-1.41)	(0.81-0.99)	(1.24-1.46)	(0.85-1.01)	(1.21-1.41)	(0.84-1.00)	(1.21-1.42)	(0.82-1.00)	(1.24-1.46)
Holland-Midden	0.92	1.23	0.96	1.17	1.00	1.12	0.94	1.19	0.96	1.17
	(0.84-1.00)	(1.15-1.32)	(0.88-1.05)	(1.08-1.25)	(0.93-1.09)	(1.04-1.21)	(0.87-1.02)	(1.11-1.29)	(0.88-1.06)	(1.09-1.26)
Hart voor Brabant	0.94	1.07	1.03	1.03	1.13	1.03	1.02	0.99	1.05	1.03
	(0.80-1.11)	(1.00-1.15)	(0.82-1.30)	(0.96-1.12)	(0.86-1.49)	(0.95-1.11)	(0.88-1.19)	(0.92-1.07)	(0.82-1.33)	(0.96-1.12)
Haaglanden	0.99	1.05	1.07	0.95	1.15	0.93	1.09	0.97	1.07	0.96
	(0.89-1.10)	(0.98-1.14)	(0.96-1.19)	(0.88-1.04)	(1.03-1.29)	(0.85-1.01)	(0.99-1.19)	(0.89-1.05)	(0.96-1.20)	(0.88-1.05)
Groningen	0.93	1.41	0.98	1.28	1.02	1.24	0.98	1.31	1.00	1.27
	(0.83-1.04)	(1.31-1.53)	(0.87-1.11)	(1.17-1.39)	(0.91-1.14)	(1.14-1.35)	(0.87-1.09)	(1.20-1.43)	(0.88-1.13)	(1.16-1.38)
Gooi en	0.95	1.15	0.97	1.18	1.07	1.13	0.99	1.12	0.98	1.17
Vechtstreek	(0.85-1.07)	(1.04-1.27)	(0.85-1.10)	(1.06-1.31)	(0.94-1.22)	(1.02-1.26)	(0.90-1.10)	(1.01-1.25)	(0.86-1.10)	(1.06-1.30)
Gelderland-Zuid	1.02	1.38	1.10	1.28	1.14	1.23	1.06	1.25	1.09	1.28
	(0.89-1.17)	(1.28-1.49)	(0.93-1.31)	(1.18-1.39)	(0.95-1.38)	(1.14-1.34)	(0.95-1.19)	(1.15-1.36)	(0.94-1.25)	(1.18-1.39)

Gelderland-Midden	0.88	1.22	0.90	1.15	0.98	1.11	0.94	1.17	0.91	1.16
	(0.79-0.97)	(1.11-1.34)	(0.81-1.01)	(1.04-1.27)	(0.87-1.10)	(1.01-1.23)	(0.85-1.04)	(1.06-1.29)	(0.81-1.01)	(1.05-1.28)
Friesland	0.94	1.33	0.96	1.38	1.05	1.32	0.98	1.35	0.98	1.37
	(0.84-1.05)	(1.24-1.44)	(0.84-1.10)	(1.27-1.50)	(0.92-1.21)	(1.22-1.43)	(0.89-1.09)	(1.24-1.47)	(0.85-1.12)	(1.27-1.49)
Flevoland	0.86	1.11	0.91	0.97	0.99	0.95	0.92	0.98	0.91	0.97
	(0.74-0.99)	(0.94-1.31)	(0.79-1.05)	(0.80-1.18)	(0.85-1.15)	(0.78-1.15)	(0.80-1.06)	(0.81-1.20)	(0.79-1.05)	(0.80-1.18)
Drenthe	0.89	1.10	0.89	1.16	0.97	1.11	0.95	1.13	0.90	1.15
	(0.77-1.04)	(0.99-1.23)	(0.76-1.03)	(1.03-1.30)	(0.86-1.10)	(0.99-1.24)	(0.84-1.08)	(1.00-1.27)	(0.77-1.05)	(1.02-1.29)
Brabant-Zuidoost	0.96	1.09	0.99	1.05	0.98	1.06	1.02	1.01	0.99	1.06
	(0.88-1.05)	(1.01-1.17)	(0.89-1.10)	(0.97-1.14)	(0.90-1.06)	(0.98-1.14)	(0.94-1.11)	(0.93-1.09)	(0.89-1.10)	(0.98-1.14)
Amsterdam	0.93	1.33	1.08	1.10	1.10	1.04	1.10	1.05	1.06	1.09
	(0.81-1.07)	(1.23-1.44)	(0.91-1.28)	(1.01-1.21)	(0.98-1.23)	(0.95-1.14)	(0.96-1.26)	(0.96-1.15)	(0.92-1.23)	(0.99-1.19)

Table A7 continued.

IRR (95%CI)	Model 4		Model 5a		Model 5b		Model 5c		Model 6	
		Infl.		Infl.		Infl.		Infl.		Infl.
Zuid-Limburg	1.00 (ref)	1.00 (ref)	1.00 (ref)	1.00 (ref)	1.00 (ref)	1.00 (ref)	1.00 (ref)	1.00 (ref)	1.00 (ref)	1.00 (ref)
Zuid-Holland-Zuid	0.95	1.05	0.95	1.05	0.95	1.05	0.95	1.05	0.94	1.06
	(0.87-1.03)	(0.95-1.16)	(0.87-1.03)	(0.95-1.17)	(0.87-1.03)	(0.95-1.17)	(0.87-1.03)	(0.95-1.16)	(0.87-1.02)	(0.96-1.17)
Zeeland	0.95	1.05	0,97	1,05	0,96	1,05	0,94	1,05	0,96	1,05
	(0.87-1.05)	(0.95-1.16)	(0,89-1,07)	(0,95-1,16)	(0,87-1,05)	(0,95-1,16)	(0,86-1,03)	(0,95-1,16)	(0,88-1,06)	(0,95-1,16)
Zaanstreek-Waterland	1,03	1,06	1,04	1,06	1,02	1,07	1,03	1,06	1,03	1,07
	(0,95-1,12)	(0,97-1,15)	(0,97-1,13)	(0,97-1,16)	(0,95-1,11)	(0,98-1,16)	(0,96-1,12)	(0,97-1,15)	(0,96-1,11)	(0,98-1,17)
West-Brabant	0,94	0,97	0,95	0,97	0,94	0,97	0,94	0,96	0,94	0,97
	(0,87-1,02)	(0,88-1,06)	(0,88-1,03)	(0,88-1,06)	(0,87-1,02)	(0,88-1,06)	(0,87-1,01)	(0,88-1,06)	(0,87-1,01)	(0,89-1,06)
Utrecht	1,05	1,12	1,05	1,12	1,04	1,12	1,04	1,12	1,04	1,13
	(0,96-1,13)	(1,04-1,20)	(0,97-1,14)	(1,05-1,20)	(0,96-1,13)	(1,05-1,20)	(0,96-1,13)	(1,04-1,20)	(0,96-1,13)	(1,05-1,21)
Twente	1,05	1,02	1,07	1,03	1,05	1,03	1,05	1,02	1,06	1,04
	(0,95-1,17)	(0,93-1,12)	(0,97-1,19)	(0,94-1,13)	(0,95-1,17)	(0,94-1,13)	(0,95-1,17)	(0,93-1,12)	(0,96-1,18)	(0,94-1,14)
Rotterdam-Rijnmond	1,09	1,02	1,10	1,02	1,09	1,02	1,09	1,02	1,09	1,02
	(1,01-1,18)	(0,94-1,09)	(1,02-1,19)	(0,95-1,10)	(1,01-1,18)	(0,95-1,10)	(1,01-1,18)	(0,94-1,09)	(1,01-1,18)	(0,95-1,10)
Noord- en Oost-Gelderland	0,99	1,22	1,00	1,23	0,98	1,22	0,99	1,22	0,98	1,23
	(0,92-1,07)	(1,13-1,32)	(0,93-1,08)	(1,13-1,33)	(0,91-1,06)	(1,13-1,33)	(0,92-1,06)	(1,12-1,32)	(0,91-1,05)	(1,14-1,34)
Limburg-Noord	1,05	0,96	1,06	0,97	1,04	0,96	1,04	0,96	1,05	0,97
	(0,93-1,18)	(0,89-1,05)	(0,94-1,20)	(0,89-1,05)	(0,93-1,17)	(0,89-1,05)	(0,93-1,18)	(0,89-1,05)	(0,93-1,18)	(0,89-1,05)
Kennemerland	1,07	1,07	1,09	1,08	1,07	1,08	1,07	1,07	1,08	1,09
	(0,98-1,17)	(0,99-1,17)	(0,99-1,19)	(1,00-1,18)	(0,98-1,17)	(0,99-1,17)	(0,98-1,17)	(0,99-1,17)	(0,99-1,18)	(1,00-1,18)
IJsselland	1,12	1,29	1,12	1,30	1,11	1,30	1,11	1,29	1,1	1,31
	(1,01-1,23)	(1,17-1,42)	(1,02-1,23)	(1,18-1,44)	(1,01-1,22)	(1,17-1,43)	(1,01-1,22)	(1,17-1,42)	(1,01-1,21)	(1,19-1,45)
Hollands Noorden	0,93	1,30	0,95	1,30	0,93	1,30	0,94	1,30	0,95	1,31
	(0,86-1,01)	(1,20-1,41)	(0,87-1,03)	(1,20-1,41)	(0,86-1,00)	(1,20-1,41)	(0,87-1,02)	(1,20-1,41)	(0,88-1,03)	(1,21-1,42)
Holland-Midden	0,98	1,17	0,99	1,18	0,97	1,17	0,97	1,17	0,97	1,18
	(0,91-1,05)	(1,08-1,26)	(0,92-1,06)	(1,09-1,27)	(0,90-1,05)	(1,09-1,26)	(0,90-1,04)	(1,08-1,26)	(0,90-1,04)	(1,10-1,27)
Hart voor Brabant	1,07	1,00	1,07	1,00	1,07	1,00	1,08	1,00	1,06	1,00
	(0,89-1,30)	(0,92-1,08)	(0,90-1,26)	(0,92-1,08)	(0,89-1,29)	(0,93-1,08)	(0,88-1,32)	(0,92-1,08)	(0,89-1,26)	(0,93-1,08)
Haaglanden	1,13	0,95	1,15	0,96	1,13	0,96	1,13	0,95	1,15	0,96
	(1,03-1,24)	(0,87-1,04)	(1,04-1,26)	(0,88-1,04)	(1,03-1,24)	(0,88-1,04)	(1,03-1,24)	(0,87-1,04)	(1,04-1,27)	(0,88-1,05)
Groningen	1,00	1,29	1,01	1,30	1,00	1,30	1,00	1,29	1,00	1,31
	(0,90-1,12)	(1,19-1,41)	(0,91-1,13)	(1,20-1,42)	(0,89-1,11)	(1,19-1,42)	(0,90-1,12)	(1,19-1,41)	(0,90-1,11)	(1,20-1,43)
Gooi en Vechtstreek	1,04	1,11	1,05	1,10	1,03	1,12	1,05	1,11	1,04	1,11
	(0,94-1,16)	(1,00-1,23)	(0,94-1,18)	(0,99-1,22)	(0,93-1,15)	(1,00-1,24)	(0,94-1,17)	(1,00-1,23)	(0,93-1,16)	(1,00-1,23)
Gelderland-Zuid	1,11	1,24	1,12	1,24	1,10	1,24	1,11	1,24	1,11	1,24
	(0,96-1,27)	(1,14-1,35)	(0,97-1,30)	(1,14-1,34)	(0,96-1,26)	(1,14-1,35)	(0,96-1,27)	(1,14-1,34)	(0,96-1,30)	(1,14-1,35)
Gelderland-Midden	0,98	1,15	0,99	1,16	0,97	1,16	0,98	1,15	0,98	1,16

	(0,88-1,08)	(1,04-1,27)	(0,89-1,09)	(1,05-1,28)	(0,88-1,07)	(1,05-1,28)	(0,88-1,09)	(1,04-1,27)	(0,88-1,08)	(1,05-1,29)
Friesland	1,03	1,33	1,04	1,33	1,02	1,33	1,03	1,33	1,03	1,34
	(0,93-1,15)	(1,22-1,44)	(0,94-1,15)	(1,23-1,45)	(0,92-1,13)	(1,23-1,45)	(0,93-1,14)	(1,22-1,44)	(0,93-1,13)	(1,23-1,45)
Flevoland	0,96	0,97	0,97	0,97	0,96	0,97	0,97	0,97	0,97	0,98
	(0,83-1,11)	(0,79-1,18)	(0,84-1,11)	(0,80-1,19)	(0,83-1,11)	(0,80-1,19)	(0,84-1,12)	(0,79-1,18)	(0,83-1,12)	(0,80-1,19)
Drenthe	0,99	1,11	1,00	1,12	0,99	1,12	0,99	1,11	0,99	1,12
	(0,88-1,11)	(0,99-1,25)	(0,89-1,12)	(0,99-1,26)	(0,88-1,11)	(0,99-1,26)	(0,88-1,11)	(0,99-1,25)	(0,89-1,11)	(1,00-1,26)
Brabant-Zuidoost	1,01	1,02	1,03	1,02	1,00	1,03	1,00	1,02	1,01	1,02
	(0,93-1,09)	(0,94-1,11)	(0,95-1,11)	(0,94-1,11)	(0,93-1,08)	(0,95-1,11)	(0,92-1,08)	(0,94-1,11)	(0,93-1,09)	(0,95-1,11)
Amsterdam	1,12	1,03	1,14	1,04	1,11	1,03	1,12	1,03	1,13	1,05
	(1,00-1,25)	(0,94-1,13)	(1,01-1,27)	(0,95-1,14)	(0,99-1,25)	(0,94-1,13)	(1,00-1,25)	(0,94-1,13)	(1.00-1,27)	(0,96-1,15)

IRR: Incidence Rate Ratio. CI: confidence interval. Model 1: region. Model 2: region, demographic factors, and SES. Model 3a: region, demographic factors, SES, and self-rated health. Model 3b: region, demographic factors, SES and chronic disease. Model 3c: region, demographic factors, SES and psychological distress. Model 4: region, demographic factors, SES, self-rated health, chronic disease, and psychological distress. Model 5a: region, demographic factors, SES, self-rated health, chronic disease, psychological distress, and lifestyle. Model 5b: region, demographic factors, SES, self-rated health, chronic disease, psychological distress, and loneliness. Model 5c: region, demographic factors, SES, self-rated health, chronic disease, psychological distress, and mastery. Model 6: region, demographic factors, SES, self-rated health, chronic disease, psychological distress, lifestyle, loneliness, and mastery. Registry data: age, gender, migration background and household income. Self-reported data: marital status, education, income inadequacy, self-rated health, chronic disease, psychological distress, lifestyle, loneliness, and mastery.

Table A8. Explanation of significant regional differences in GP consult costs by adding lifestyle, loneliness and mastery (model 4 compared to model 6).

	Model 4		Model 6				
Region	marginal costs	Difference with ref	marginal costs	Difference with ref	Difference between model 4 and 6	Average number of citizens aged 19 or older in 2017*	Increase or decrease regional differences in costs
Zuid-Limburg	47,98	(ref)	€ 47,86	(ref)			
Zuid-Holland-Zuid	€ 42,61	€ 5,37	€ 42,41	€ 5,45	€ 0,08	334.140	€ 26.346,94
Zeeland	€ 39,21	€ 8,77	€ 38,99	€ 8,87	€ 0,10	306.222	€ 30.282,29
Zaanstreek-Waterland	€ 42,61	€ 5,37	€ 42,54	€ 5,32	€ -0,05	264.893	-€ 12.638,05
West-Brabant	€ 38,84	€ 9,14	€ 38,73	€ 9,13	€ -0,01	562.773	-€ 7.203,49
Utrecht	€ 41,16	€ 6,82	€ 41,23	€ 6,63	€ -0,19	666.844	-€ 127.840,66
Twente	€ 36,71	€ 11,27	€ 36,78	€ 11,08	€ -0,19	494.646	-€ 93.859,08
Rotterdam-Rijnmond	€ 37,55	€ 10,43	€ 37,57	€ 10,29	€ -0,14	1.033.491	-€ 148.636,68
Noord- en Oost-Gelderland	€ 37,92	€ 10,06	€ 37,82	€ 10,04	€ -0,02	649.777	-€ 13.002,04
Limburg-Noord	€ 44,02	€ 3,96	€ 43,97	€ 3,89	€ -0,07	421.842	-€ 29.621,75
Kennemerland	€ 41,58	€ 6,40	€ 41,72	€ 6,14	€ -0,26	421.650	-€ 109.430,82
IJsselland	€ 39,04	€ 8,94	€ 38,89	€ 8,97	€ 0,03	403.397	€ 11.258,81
Hollands Noorden	€ 38,80	€ 9,18	€ 39,01	€ 8,85	€ -0,33	518.725	-€ 170.183,30
Holland-Midden	€ 40,03	€ 7,95	€ 39,80	€ 8,06	€ 0,11	551.554	€ 61.277,65
Hart voor Brabant	€ 43,74	€ 4,24	€ 43,88	€ 3,98	€ -0,26	843.239	-€ 219.958,89
Haaglanden	€ 32,89	€ 15,09	€ 32,92	€ 14,94	€ -0,15	860.697	-€ 132.194,45
Groningen	€ 41,70	€ 6,28	€ 41,73	€ 6,13	€ -0,15	475.810	-€ 73.545,95
Gooi en Vechtstreek	€ 43,18	€ 4,80	€ 43,48	€ 4,37	€ -0,43	197.886	-€ 84.410,25
Gelderland-Zuid	€ 42,73	€ 5,25	€ 42,68	€ 5,18	€ -0,07	440.023	-€ 31.611,25
Gelderland-Midden	€ 40,59	€ 7,39	€ 40,45	€ 7,41	€ 0,02	537.356	€ 9.618,67
Friesland	€ 39,12	€ 8,86	€ 39,07	€ 8,78	€ -0,08	511.208	-€ 38.524,63
Flevoland	€ 42,20	€ 5,78	€ 42,25	€ 5,60	€ -0,18	391.288	-€ 69.023,20
Drenthe	€ 39,87	€ 8,11	€ 39,98	€ 7,88	€ -0,23	601.197	-€ 139.736,22
Brabant-Zuidoost	€ 41,20	€ 6,78	€ 41,36	€ 6,50	€ -0,28	847.533	-€ 239.097,53
Total							-€ 1.601.733,89

Model 4: region, demographic factors, SES, self-rated health, chronic disease and psychological distress. Model 6: region, demographic factors, SES, self-rated health, chronic disease, psychological distress, lifestyle, loneliness, and mastery. Registry data: age, gender, migration background and household income. Self-reported data: marital status, education, income inadequacy, self-rated health, chronic disease, psychological distress, lifestyle, loneliness and mastery. * Based on data provided by Statistics Netherlands (opendata.cbs.nl)

Table A9. Explanation of significant regional differences in mental healthcare costs by adding lifestyle, loneliness and mastery (model 4 compared to model 6).

	Model 4		Model 6				
Region	marginal costs	Difference with ref	marginal costs	Difference with ref	Difference between model 4 and 6	Average number of citizens aged 19 or older in 2017*	Increase or decrease regional differences in costs
Zuid-Limburg	€ 215,39	(ref)	€ 212,84	(ref)			
Twente	€ 135,08	€ 80,31	€ 138,40	€ 74,44	-€ 5,87	494.646	-€ 2.903.572,02
Hollands Midden	€ 138,53	€ 76,86	€ 137,70	€ 75,14	-€ 1,72	551.554	-€ 948.672,88
Zuid-Holland-Zuid	€ 107,34	€ 108,05	€ 105,40	€ 107,44	-€ 0,61	334.140	-€ 203.825,40
Total							-€ 4.056.070,30

Model 4: region, demographic factors, SES, self-rated health, chronic disease and psychological distress. Model 6: region, demographic factors, SES, self-rated health, chronic disease, psychological distress, lifestyle, loneliness, and mastery. Registry data: age, gender, migration background and household income. Self-reported data: marital status, education, income inadequacy, self-rated health, chronic disease, psychological distress, lifestyle, loneliness and mastery. * Based on data provided by Statistics Netherlands (opendata.cbs.nl)

Figure A1. Marginal costs of region for GP consult costs based on unadjusted (model 1) and fully adjusted costs (model 6).

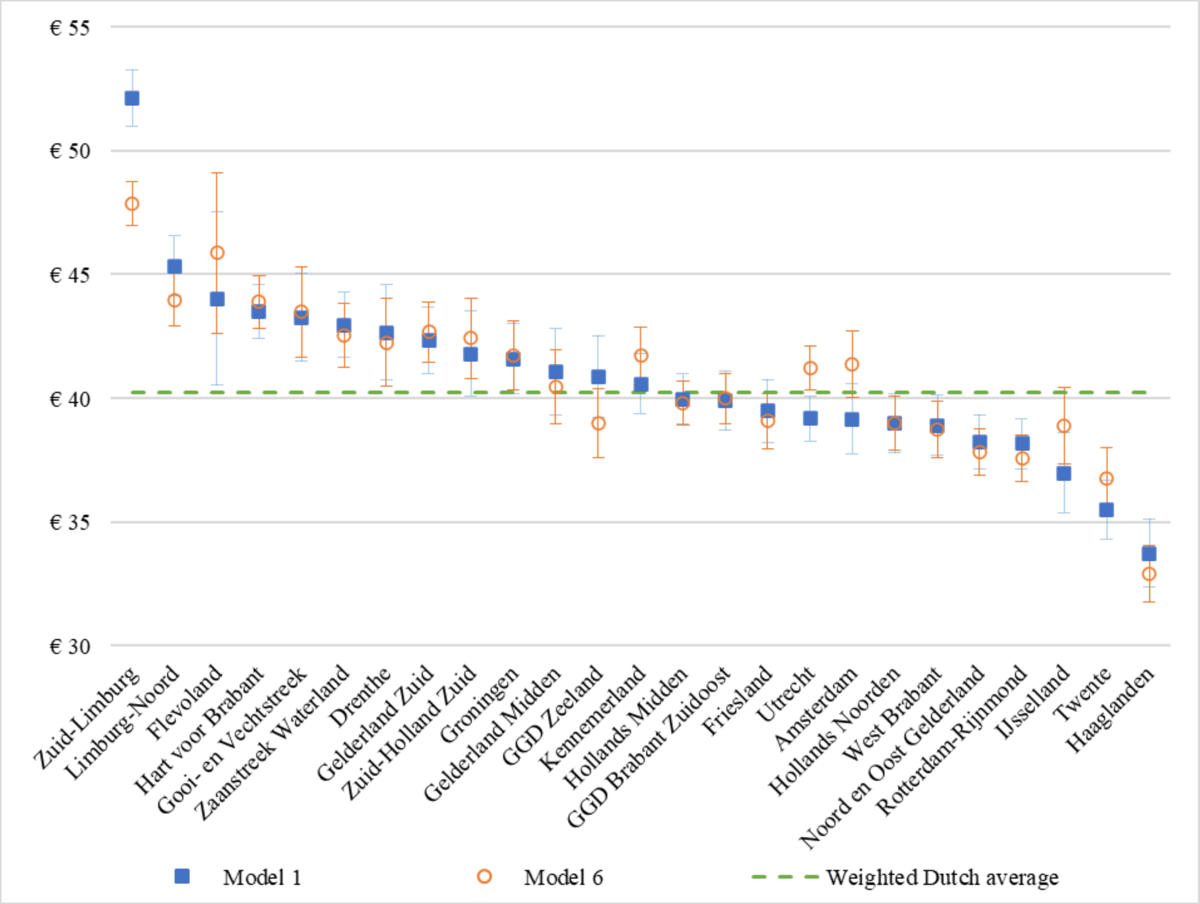

* Model 1 is adjusted for region only. In model 6, the association of region and healthcare costs is adjusted demographic factors, SES, general and mental health, lifestyle, loneliness and mastery. Registry data: age, gender, migration background and household income. Self-reported data: marital status, education, income inadequacy, self-rated health, chronic disease, psychological distress, lifestyle, loneliness and mastery.

Figure A2. Marginal costs of region for mental healthcare costs based on unadjusted (model 1) and fully adjusted costs (model 6).

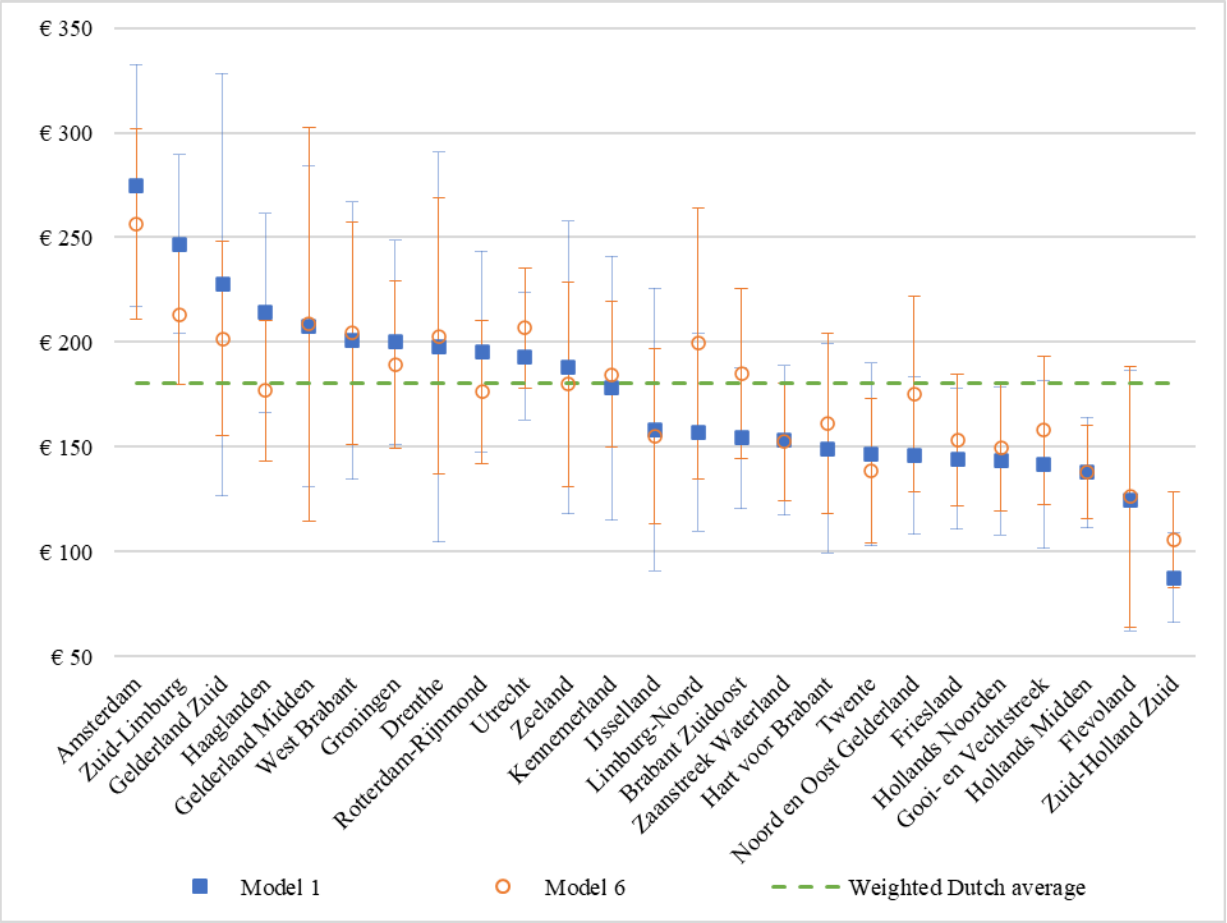

* Model 1 is adjusted for region only. In model 6, the association of region and healthcare costs is adjusted demographic factors, SES, general and mental health, lifestyle, loneliness and mastery. Registry data: age, gender, migration background and household income. Self-reported data: marital status, education, income inadequacy, self-rated health, chronic disease, psychological distress, lifestyle, loneliness and mastery.

Figure A3. Marginal costs of region for pharmaceutical costs based on unadjusted (model 1) and fully adjusted costs (model 6).

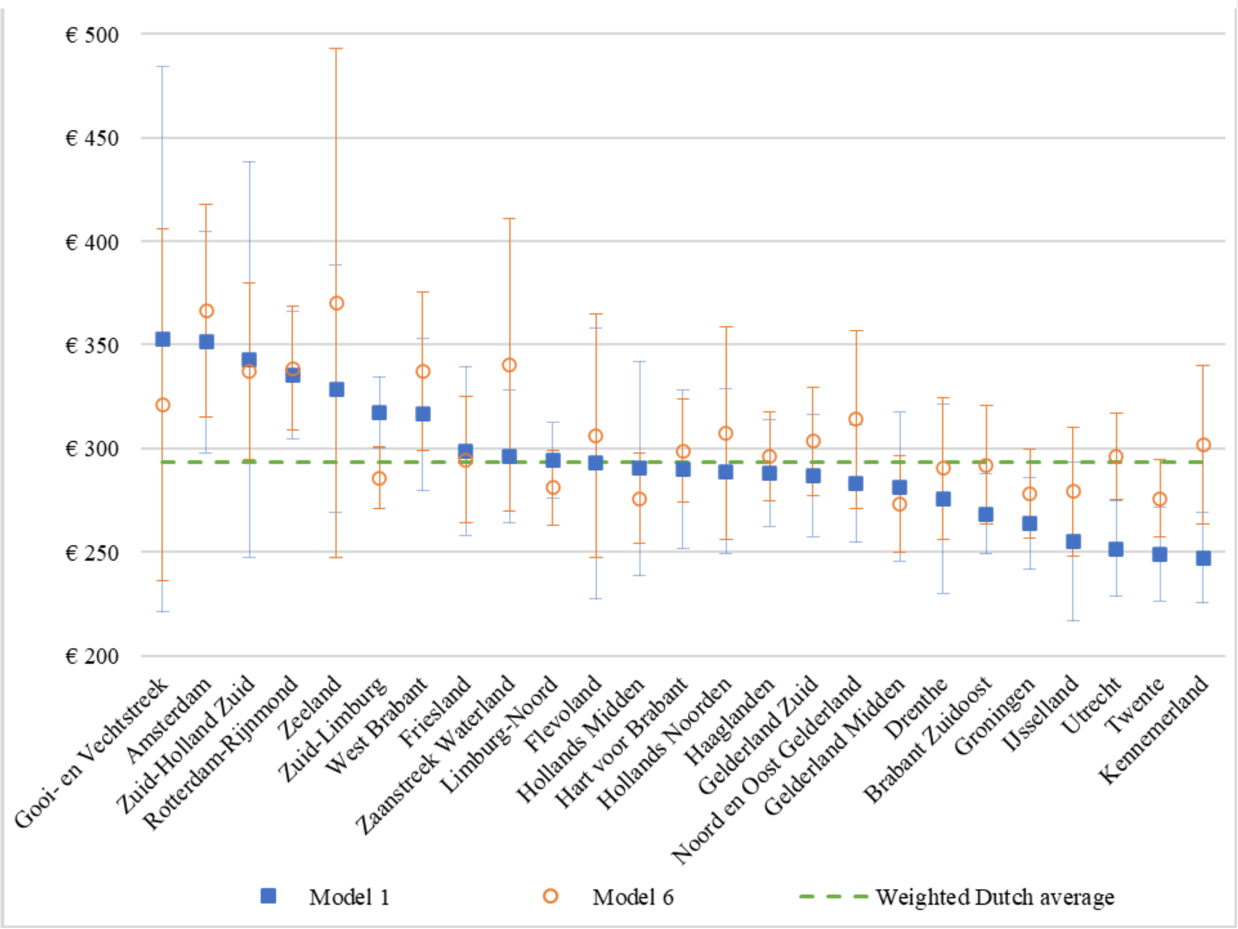

* Model 1 is adjusted for region only. In model 6, the association of region and healthcare costs is adjusted demographic factors, SES, general and mental health, lifestyle, loneliness and mastery. Registry data: age, gender, migration background and household income. Self-reported data: marital status, education, income inadequacy, self-rated health, chronic disease, psychological distress, lifestyle, loneliness and mastery.

Figure A4. Marginal costs of region for specialized care costs based on unadjusted (model 1) and fully adjusted costs (model 6).

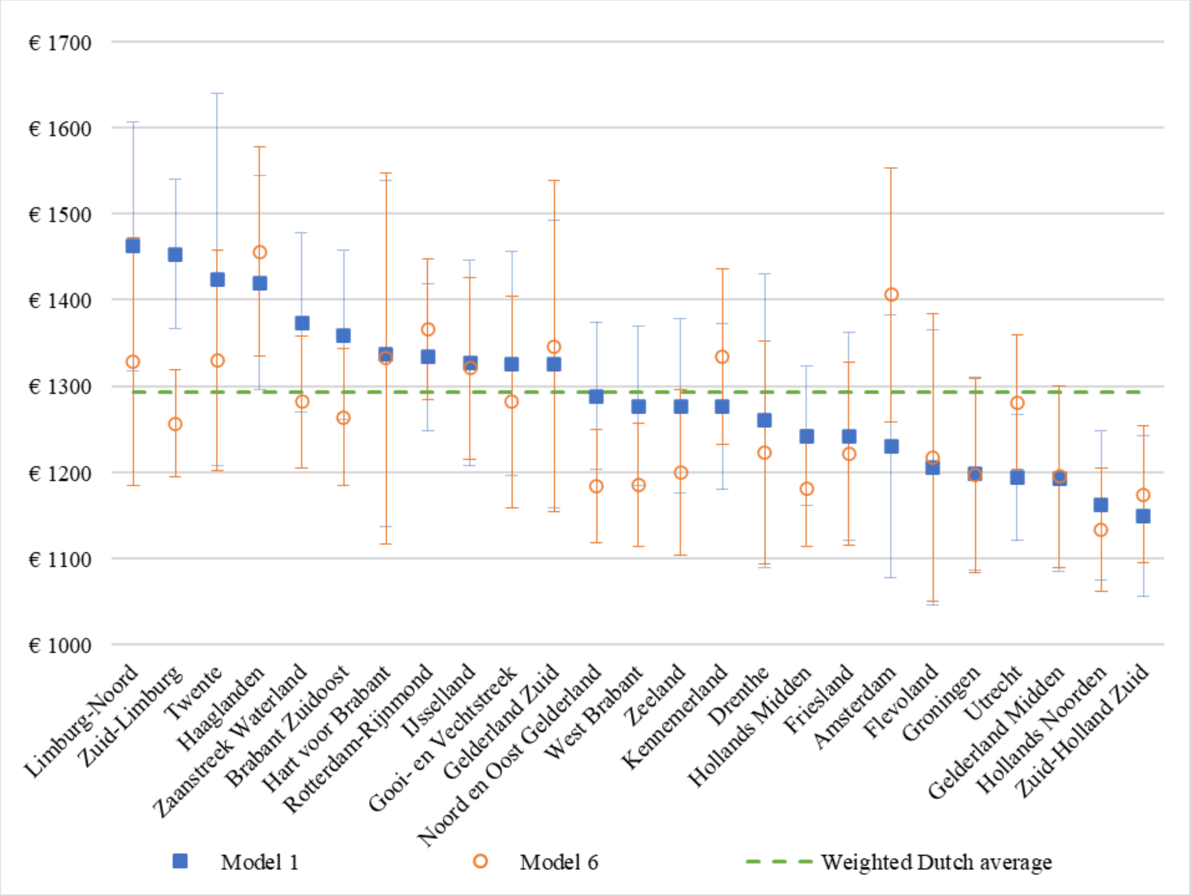

* Model 1 is adjusted for region only. In model 6, the association of region and healthcare costs is adjusted demographic factors, SES, general and mental health, lifestyle, loneliness and mastery. Registry data: age, gender, migration background and household income. Self-reported data: marital status, education, income inadequacy, self-rated health, chronic disease, psychological distress, lifestyle, loneliness and mastery.
